# Supplementary material for: Ions Modulate Stress-Induced Nanotexture in Supported Fluid Lipid Bilayers
Source: Biophys J. 2017 Jul 25;113(2):426–39. doi: 10.1016/j.bpj.2017.05.049 (PMC5529180; doi:10.1016/j.bpj.2017.05.049)
Supplement: Document S1. Supporting Materials and Methods and Figs. S1–S12 [file mmc1.pdf]

Biophysical Journal, Volume *113*

Supplemental Information

Ions Modulate Stress-Induced Nanotexture in Supported Fluid Lipid Bilayers

Luca Piantanida, Hannah L. Bolt, Neshat Rozatian, Steven L. Cobb, and Kislon Voïtchovsky

Supporting Material

Ions modulate stress-induced nano-texture in supported fluid lipid bilayers

Luca Piantanida[1], Hannah L. Bolt[2], Neshat Rozatian[2], Steven L. Cobb[2], and Kislon Voïtchovsky[1*]

[1]Department of Physics and [2]Department of Chemistry, Durham University, South Road, Durham, DH1 3LE, UK.

TABLE OF CONTENT OF THE SUPPORTING MATERIAL

1. Impact of salt concentration: Deposition vs Imaging solution
2. Temporin L characterisation data
3. Influence of the tip curvature radius
4. Analysis methods for quantification of the membrane texture
5. Fluorescence recovery after photobleaching (FRAP) analysis
6. Reversibility of the tip-induced nano-texturing
7. Cholesterol structuring effect
8. Comparison of imaging modes: contact vs amplitude modulation
9. Analysis of a DOPC bilayer on silicon oxide
10. Effect of temperature on DOPC with solution 4
11. Effect of temperature on DOPS SLBs
12. Comparison of texture formation on different lipids (DOPC and POPC)
13. Supporting references

1. IMPACT OF SALT CONCENTRATION: DEPOSITION VS IMAGING SOLUTION

The saline concentration of the solution used to create the SLBs (formation solution) is 15 times higher than that of the solution used for imaging. It is well known that ions play an important role for vesicle deposition and fusion when forming SLBs (1, 5). Ions influence the lipid-lipid interactions as well as the hydration state of lipid headgroups and mediate, interactions between the proximal leaflet and the substrate. The study examines the impact of ions on the nanoscale behaviour of SLBs. To emphasise effects related to the supporting structure, we rinsed fully formed membranes with a diluted solution so as to limit ionic effects in the distal leaflet by comparison with the proximal where ions are partially trapped between the membrane and the substrate. Figure S1 shows a comparison between supported lipid bilayer formed in solution 7 (Tris 10 mM / NaCl 150 mM / $CaCl_2$ 2 mM) and imaged in four different dilutions of the deposition solution (undiluted, 5×, 10×, and 15×). Generally, the dilution does not prevent the apparition of nano-texture but emphasises the effect of tip-induced stress. At full concentration (undiluted), some texture is clearly visible in Medium imaging conditions already (see Materials and Methods for a definition of the imaging conditions). As the imaging solution is diluted, the effect becomes less intense and only occurs under Hard conditions, but its aspect remain similar in all imaging conditions. Using diluted solutions not only enhances the effect of ion-mediated interaction with the substrate, but it also enables working with strongly texturing solution (e.g. solution 6, see Fig. 1). We applied this protocol to all the imaging experiment (AFM and FRAP) in order to give a complete comparison of the data.

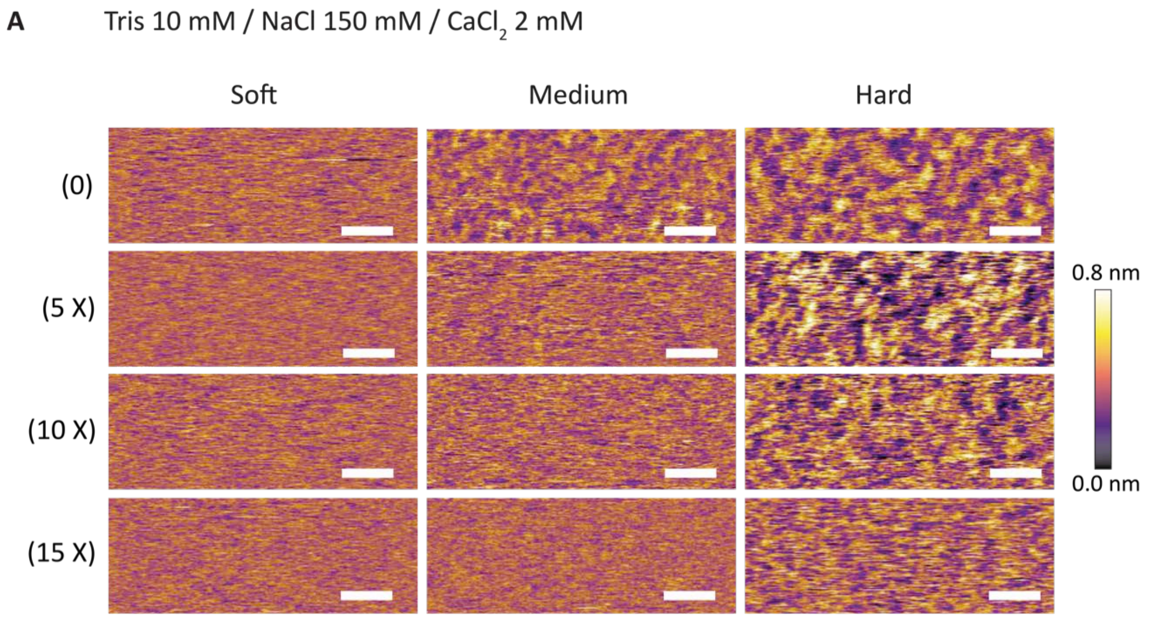

FIGURE S1. Comparison of representative AFM images of SLBs formed in different dilutions of solution 7 (Tris 10 mM / NaCl 150 mM / $CaCl_2$ 2 mM) under Soft, Medium and Hard imaging conditions (see Materials and Methods for details on the imaging parameters). The membrane is always formed in solution 7, but the sample is then rinsed with either undiluted, 5×, 10×, or 15× diluted solution 7 before the AFM measurement. Stress-induced texture is visible all cases, but its intensity decreases with diluting the solution. The scale bar is 50 nm in all images.

2. TEMPORIN L CHARACTERISATION DATA

QToF MS

Mass spectroscopy measurements were performed using a QToF Premier mass spectrometer with an Acquity ultra-performance liquid chromatography system (Waters Ltd, UK). Samples were injected to the Acquity UPLC BEH C18 column (1.7 µm, 2.1 mm x 100 mm) with a flow rate of 0.6 mL min^{-1} and a linear gradient of 0–99 % of solvent B over 6 min (A = 0.1 % formic acid in H_2O, B = 0.1 % formic acid in acetonitrile). The solvent flow from the UPLC was injected into a 0.2 mL/min flow of acetonitrile which was introduced into the electrospray ion source. Temporin L mass calculated for *m*/*z* $[M+H]^+$ 821.0, mass observed $[M+2H]^{2+}$ 820.7

Analytical HPLC

Samples were dissolved in 100 µL acidified water and the purity of products was estimated by an injection of 10 µL to analytical RP-HPLC using a Perkin Elmer 200 Series LC pump with a Perkin-Elmer 785A UV-vis detector on an SB Analytical column (ODS-H Optimal), 4.6 x 100mm , 3.5 µm; flow rate = 1 mL min^{-1}; loop size = 20 µL; column oven 40 ˚C; λ = 220 nm; gradient: 0–100 % solvent B over 30 min (solvent A: 95 % H_2O, 5 %, MeCN, 0.05 % TFA; solvent B: 95 %, MeCN, 5 % H_2O, 0.03 % TFA). Temporin L was found by RP-analytical HPLC RT 17.8 min with approximate purity > 99 %.

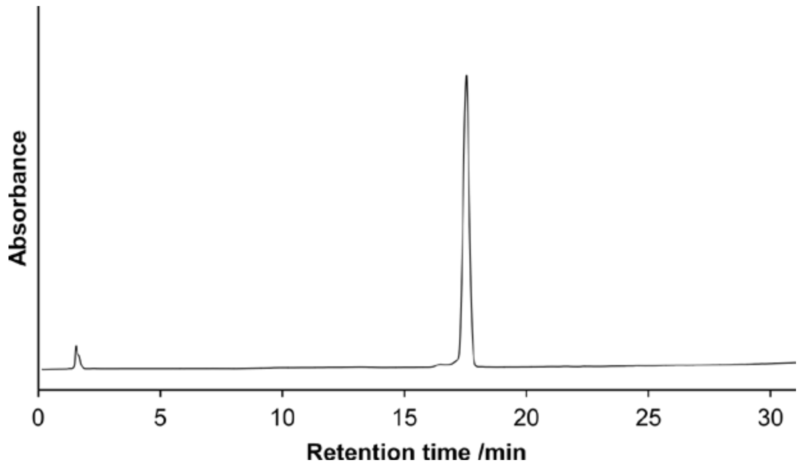

FIGURE S2. Analytical RP-HPLC chromatogram for Temporin L peptide. Flow rate = 1 mL min^{-1}; loop size = 20 µL; column oven 40 ˚C; λ = 220 nm; gradient: 0–100 % solvent B over 30 min (solvent A: 95 % H_2O, 5 %, MeCN, 0.05 % TFA; solvent B: 95 %, MeCN, 5 % H_2O, 0.03 % TFA).

3. INFLUENCE OF THE TIP CURVATURE RADIUS

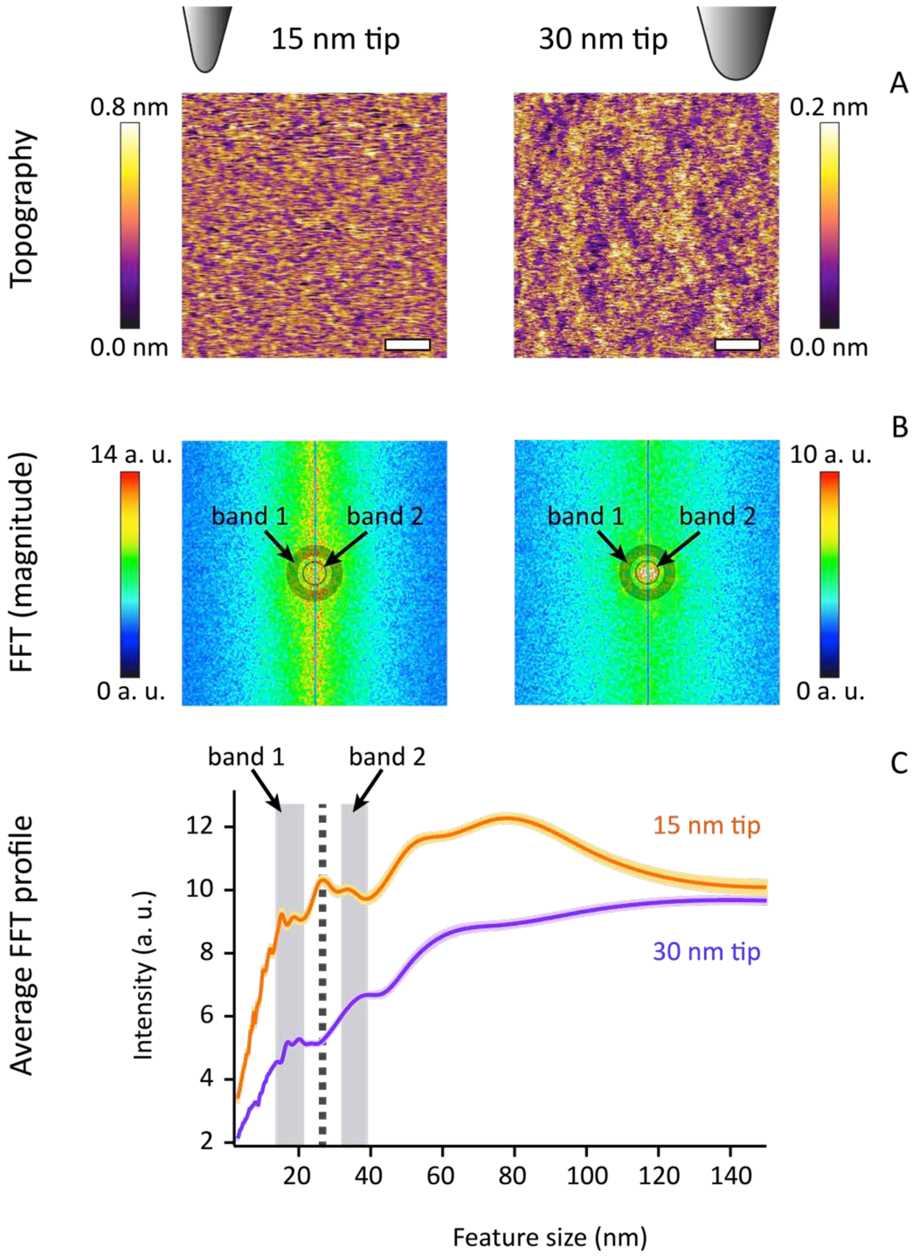

FIGURE S3. Comparison of the stress-induced features obtained with tips exhibiting different radii. The sharper tip has a maximum tip radius of 15 nm (Olympus, RC800PSA). The blunter tip is rounded by design to a 30 nm radius (Nanosensor, SD-R30-FM). Both tips are composed of silicon oxide. Topographic images were acquired in Hard imaging conditions with each tip over a same sample (A). Qualitatively, the images both exhibit some texture, but some differences are visible. First the height variations are more important with the sharper tip, reflecting the higher local pressures exerted by the tip on the membrane. To quantify similarities and differences, Fourier analysis was performed on both images (B-C). Two bands (grey area in B and C) highlight local maxima corresponding to features of size ~20 nm and ~35 nm that are present in the 2D FFT derived with both tips. This suggests that the nano-texture is an intrinsic property of the DOPC membrane on mica, rather than a reflection of the tip geometry. There are, however, some differences highlighted by the black dotted line in (C). Small differences are to be expected if the texture is induced by a local fluid to gel-like transition promoted by the tip pressure, but the existence of ~20 nm nodules suggest an ideal packing arrangement for the lipid molecules under pressure. The scale bar in (A) is 50 nm and the imaging is conducted on a DOPC bilayer immersed in solution 7 (Tris 10 mM / NaCl 150 mM / $CaCl_2$ 2 mM).

4. ANALYSIS METHODS FOR QUANTIFICATION OF THE MEMBRANE TEXTURE

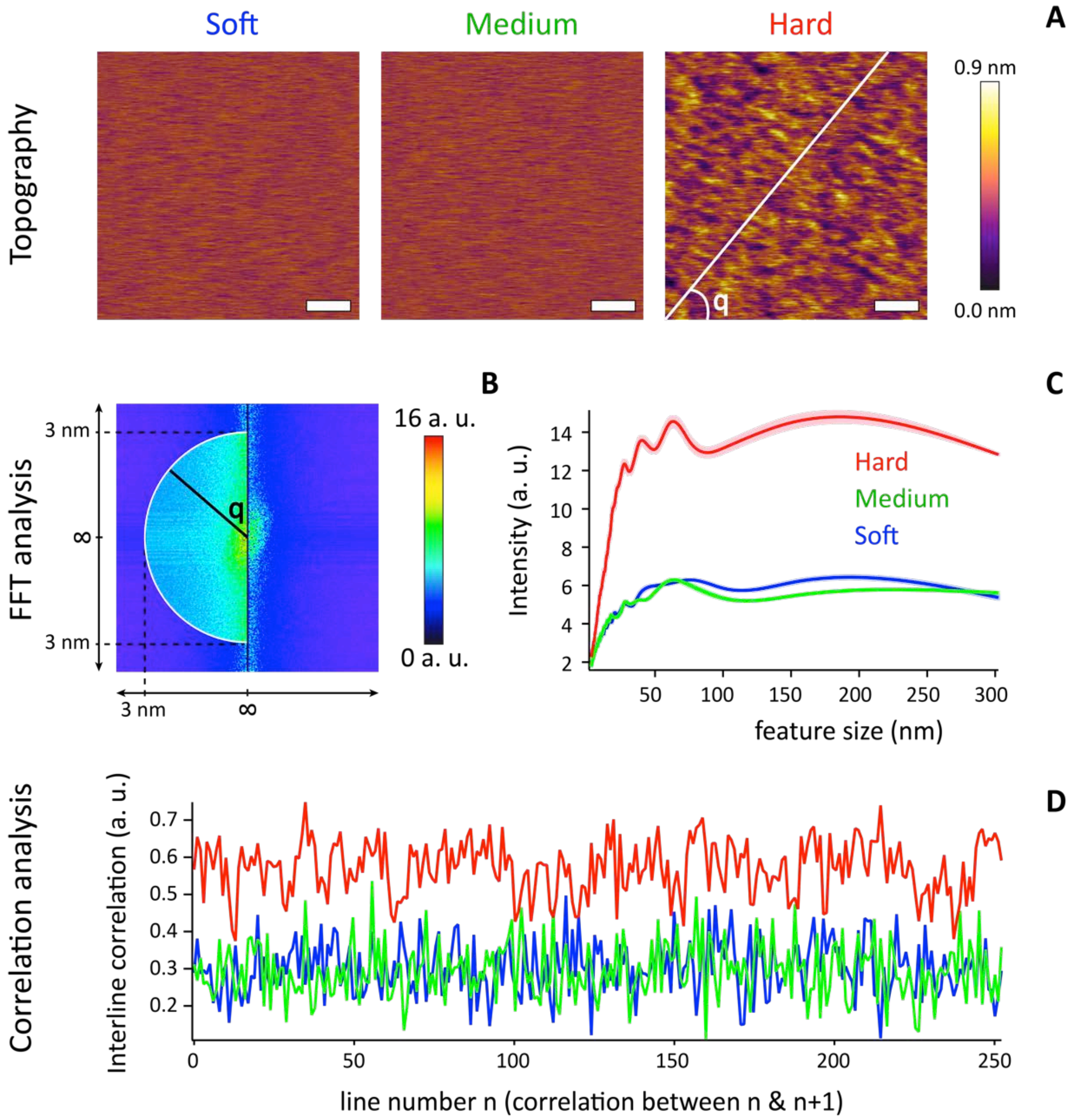

FIGURE S4. Analysis methods used to quantitatively process the stress-induced texturing of bilayers, illustrated here for DOPC immersed in solution 7 (Tris 10 mM / NaCl 150 mM / $CaCl_2$ 2 mM). Qualitatively, the topographic images (A) reveal little change between Soft and Medium imaging conditions, but a dramatic change occurs in Hard conditions. The 2D FFT of the image acquired in Hard conditions is shown in (B). The black line is the 1D FFT of the profile shown in white in (A, Hard). It covers features with sizes between 3 nm and infinity at the centre of the image (reciprocal space). The profile is then averaged over all directions (all θ angles, with 90° < θ < 270°) and the average converted back to real space (C). The converted averaged profiles are given in (C) for each imaging conditions, with the shading representing the standard error. The intensity is significantly higher for the Hard imaging conditions while the Soft and Medium conditions are similar, reflecting the qualitative observations of the height images. The quantitative increase in long-range order is subsequently calculated by integration of the Fourier profiles over a selected interval of feature sizes (see text). The correlation analysis (D) examines the degree of correlation between each set of 2 adjacent scan profiles. Since each image is composed of 256 lines, 254 pairs (correlation values) are obtained with each value between 0 (no correlation at all) and 1 (identical profiles) (curves in D). The degree of correlation is overall higher in for the Hard imaging conditions as expected from the topographic images. A single correlation value is obtained for each image by averaging the 255 correlation values and taking its standard error. The Scale bar in (A) is 50 nm.

5. FLUORESCENCE RECOVERY AFTER PHOTOBLEACHING (FRAP) ANALYSIS

The lipids diffusion coefficient was calculated for DOPC immersed in each of the solutions investigated (Table 1). The measurements were performed using FRAP analysis (see Materials and Methods). Since our bleaching pattern is a square, we adapted the derivation given in reference (2) for the fluorescence intensity $I(t)$ as follow:

$$I(t) = a_0 + a_1\left(1 - \sqrt{\frac{w^2}{w^2 + 4\pi D(t - t_{bleach})}}\right) \qquad (s1)$$

where t is time, the spot area geometry is represented by the square width w (µm) and D (µm^2/s) is the diffusion coefficient. The acquired sequence coincides with the start of fluorescence recovery where t_{bleach} is zero. Before analysis, the recovery spectra are normalised using two parameters: the offset (a_0) and the amplitude (a_1) of the recorded intensity. The diffusion coefficients used in the paper are an average of at least three different sequences captured, and the associate uncertainty corresponds to one standard deviation. Higher uncertainties are found when the SLB patches exhibit significantly size variations (Fig. S5 F).

Figure S4 shows an extract of a typical FRAP experiments. Four representative frames are displayed (Fig. S5 B-E) with sequential fluorescence recovery of the bleached region (red arrow). The fluorescence intensity of the bleached area is calculated by averaging the mean intensity value of all pixels of the area (red arrow). The fluidity of the lipids in the SLB produces fluctuations in the intensity value that can be seen in the example intensity profiles shown immediately under each frame (Fig. S5 B-E). Un-fused lipid vesicles induce the brighter background ‘spots’ in the florescence images, but this can be discarded in the analysis.

Occasionally, samples presented discontinuities with several distinct SLB patches within the experimentally accessible area (500×500 µm^2) (Fig. S5 F). FRAP measurements carried out on the smaller patches yield lower diffusion coefficients when compared with the same experiment on a complete uniform bilayer (Fig. S5 G) due to edge effects. The substrate can also impact the mobility of lipid molecules when results are compared with free standing bilayers (3). Edges effect can however be neglected when investigating nanoscale texturing effects since the imaging scale is considerably smaller.

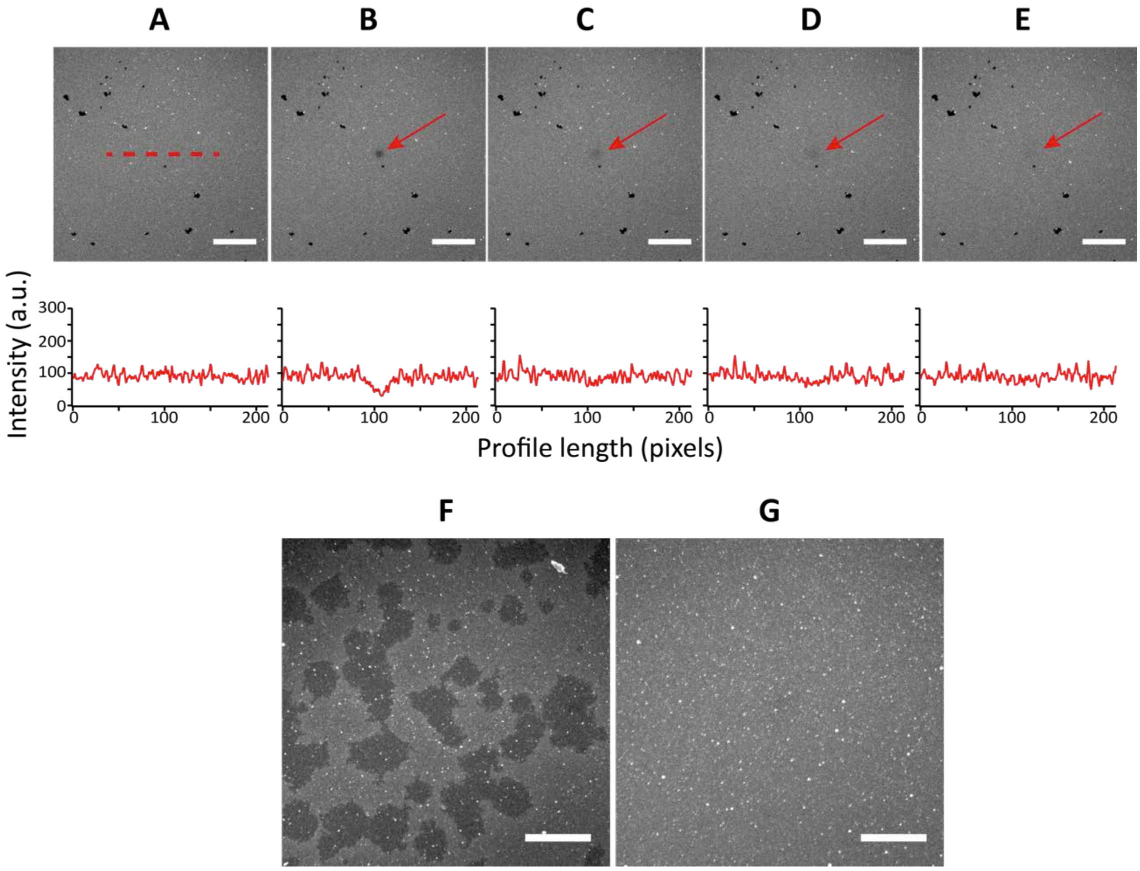

FIGURE S5. Representative fluorescence optical images of a FRAP experiment. An extract of a typical FRAP sequence: image (A) represents the analysed area with the laser off, image (B) is the first frame after bleaching with the bleached area (arrow) clearly visible (see intensity profile). Images (C, D and E) show the gradual fluorescence recovery of the area (arrow). Fluorescence intensity profiles taken across the bleached region are shown below each image. Intensity fluctuations in the profiles are due to the high lipids mobility. Images (F) and (G) illustrate two types of DOPC SLBs (both on mica in solution 7). In (F) the SLB is constituted of bilayer patches while in (G) no patches are visible suggesting a uniform bilayer. All scale bars are 100 µm.

6. REVERSIBILITY OF THE TIP-INDUCED NANO-TEXTURING

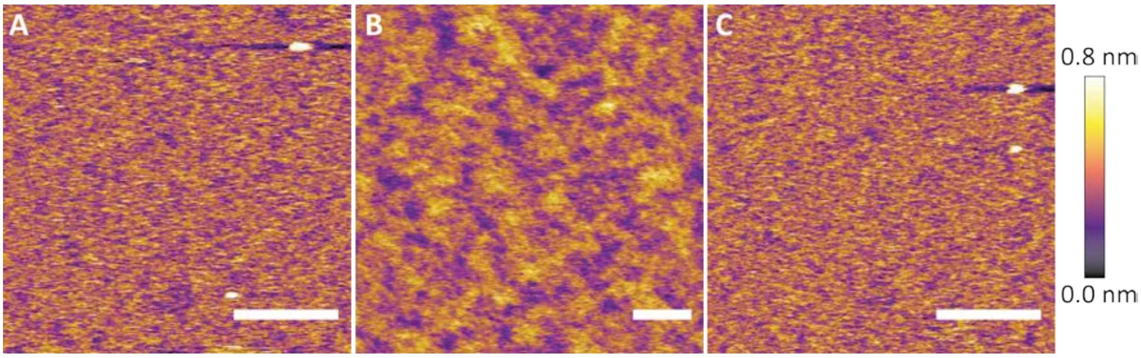

FIGURE S6. AFM images of a DOPC bilayer on mica in solution 6 (KCl 150 mM / $CaCl_2$ 2 mM). First a 1×1 μm^2 area is imaged (A) in the softest condition possible (amplitude 2 nm), followed by a zoomed in image acquired in Hard condition (300×300 nm^2, amplitude 0.6 nm) (B). This is immediately followed by a zoom out image centred over the same area, but acquired again with 2 nm amplitude (1×1 μm^2) (C). No texture or ‘memory effect’ is visible in the centre of image (C), indicating a relaxation faster than the experimental time (typically 2 minutes). This confirms a high lipid mobility. Scale bars are 300 nm (A, C) and 50 nm (B).

7. CHOLESTEROL STRUCTURING EFFECT

Cholesterol is widely used in model membrane systems due to its importance in natural cell membranes (6). The solubilisation of cholesterol into bilayers influences both fluidity and phase behaviour (4, 7). Cholesterol has a stabilising effect (8-10) on the phospholipids; it modifies the position of lipid tails, and hence the packing arrangement and the overall bilayer thickness.

Here we examine the influence of cholesterol on the stress-induced nano-textured bilayer phase transition observed by AFM. Figure S7 compares AFM images of DOPC bilayer with different concentrations of cholesterol imaged in Hard conditions. Cholesterol is not necessary for nano-texturing of the membrane (Fig. 1), but it clearly reinforces the trend (Fig. S7 C) as to be expected from a molecules promoting gel behaviour in bilayers.

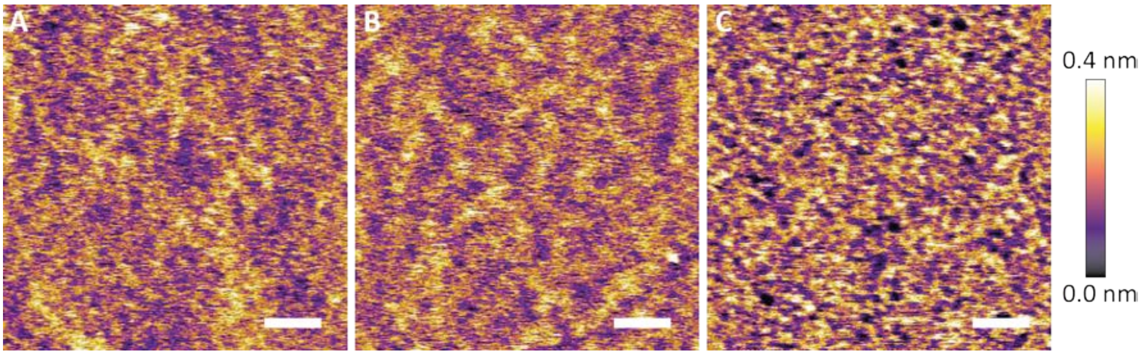

FIGURE S7. Effect of cholesterol on the nano-texturing of DOPC lipid bilayer in solution 1 (KCl 150 mM). The AFM images are taken in Hard imaging condition. Some texture is already visible when no Cholesterol is added (A), but becomes progressively more obvious as 5% relative concentration is added (B) and even more dramatically at 40% concentration (C). Image scale bars are 50 nm.

8. COMPARISON OF IMAGING MODES: CONTACT VS AMPLITUDE MODULATION

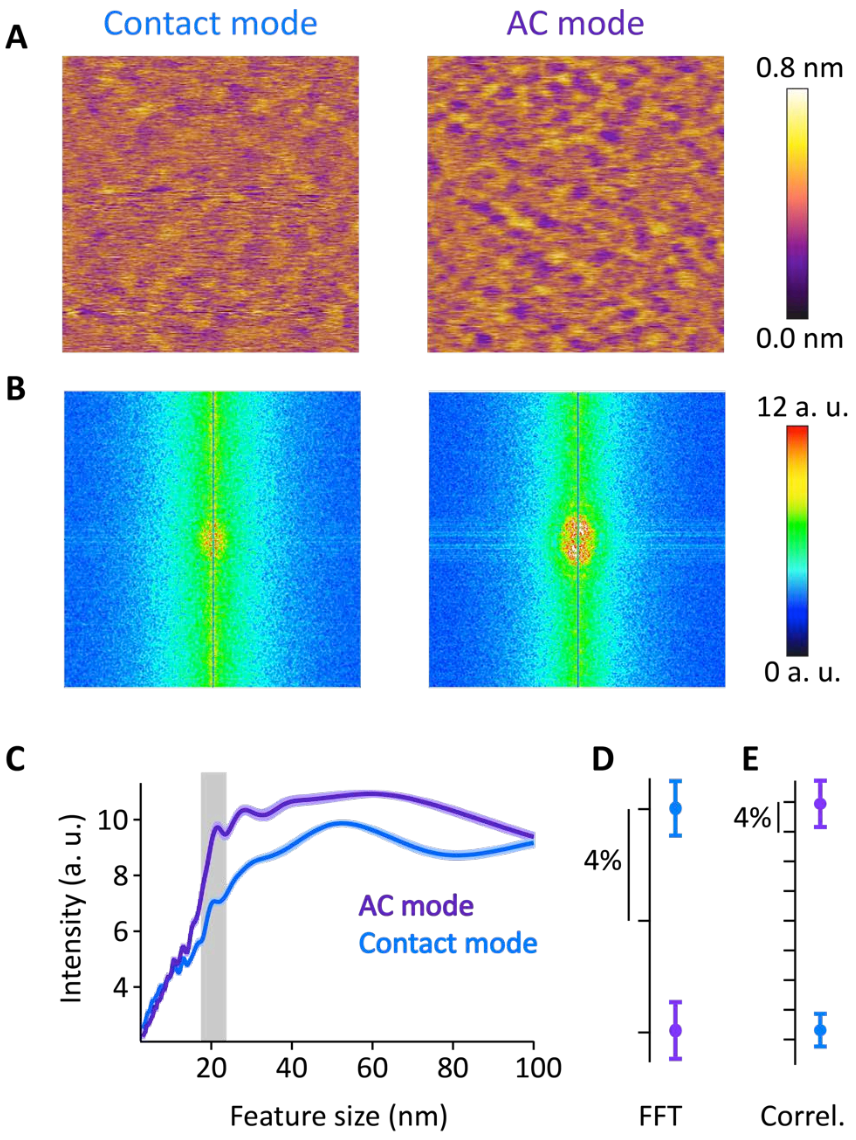

FIGURE S8. Comparison of the features derived from images obtained in contact mode and in amplitude modulation (AM). The contact mode image was obtained in the softest possible conditions, and using the same cantilever as for the AM image (A). The membrane being fluid when not stressed, the contact mode image is challenging and the tip eventually 'ploughs' through the membrane over the following images due to the shear force imposed while scanning. The AM image is obtained in Hard conditions. The 2D FFT images are shown in (B) for both images, on a same colour scale. The average profiles (C, see Fig S4 for explanations) show a maximum around 20 nm for both imaging modes (grey shading) although the maximum is significantly more marked in AM mode. There are also other maxima visible only for the AM image, consistent with a richer variety of features visible in the topographic image. Results of FFT and correlation analysis, conducted as described in the previous section, are given in (D) and (E). Since the analysis is for a single image in each case (and not a difference between Soft and Hard conditions), absolute values are given. The scale represents the different between the two modes, as percentage of the largest value. The FFT analysis (D) shows limited differences between Contact and AM modes (~8%) especially when errors are taken into account. This indicates that long range order exists in both cases. The degree of correlation is however clearly larger (~32%) for the AM images, reflecting a more solid-like membrane under imaging. The scale bar in (A) is 50 nm and the imaging is conducted on a DOPC bilayer immersed in solution 8 (Tris 10 mM / KCl 150 mM / $CaCl_2$ 2 mM).

9. ANALYSIS OF A DOPC BILAYER ON SILICON OXIDE

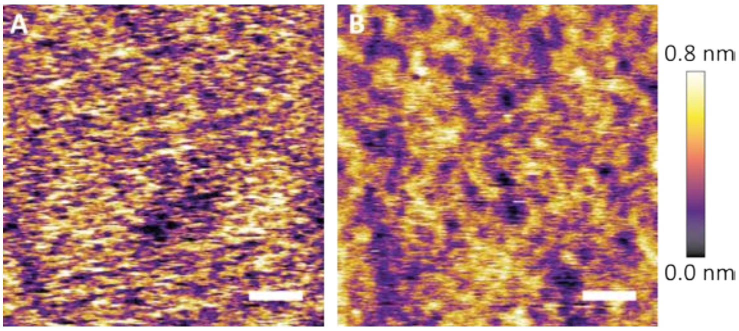

FIGURE S9. AFM analysis of a DOPC SLB formed on a silicon oxide substrate. The cleaned silicon oxide immersed in pure water (A) presents a roughness that is comparable to that of stress-induced nodules in the bilayer, prohibiting any quantitative analysis. The roughness of the DOPC SLB formed on silicon oxide is close to 1 nm, even when using Soft imaging condition (B). The scale bars are 50 nm.

10. EFFECT OF TEMPERATURE ON DOPC WITH SOLUTION 4

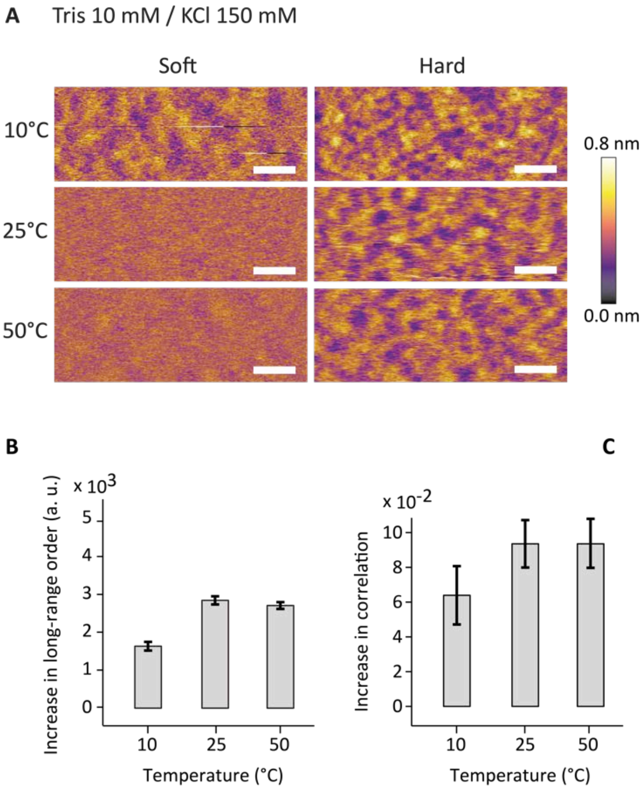

FIGURE S10. AFM images of the evolution of DOPC SLBs on mica as a function of temperature. The experimental is conducted in solution 4 (Tris 10 mM / KCl 150 mM) (A). The graphs in (B) and (C) quantify the evolution of the relative stress-induced nano-texture as a function of temperature. The existence of long-range order is given by FFT analysis (B) while line-by-line correlation (C) quantifies short-range order on the membrane (see Materials and Methods and Fig. S4 for details). In this case, a significant decrease of order is visible at 25 °C and 50 °C in Soft but not in Hard imaging condition. The images scale bar is 50 nm.

11. EFFECT OF TEMPERATURE ON DOPS SLBs

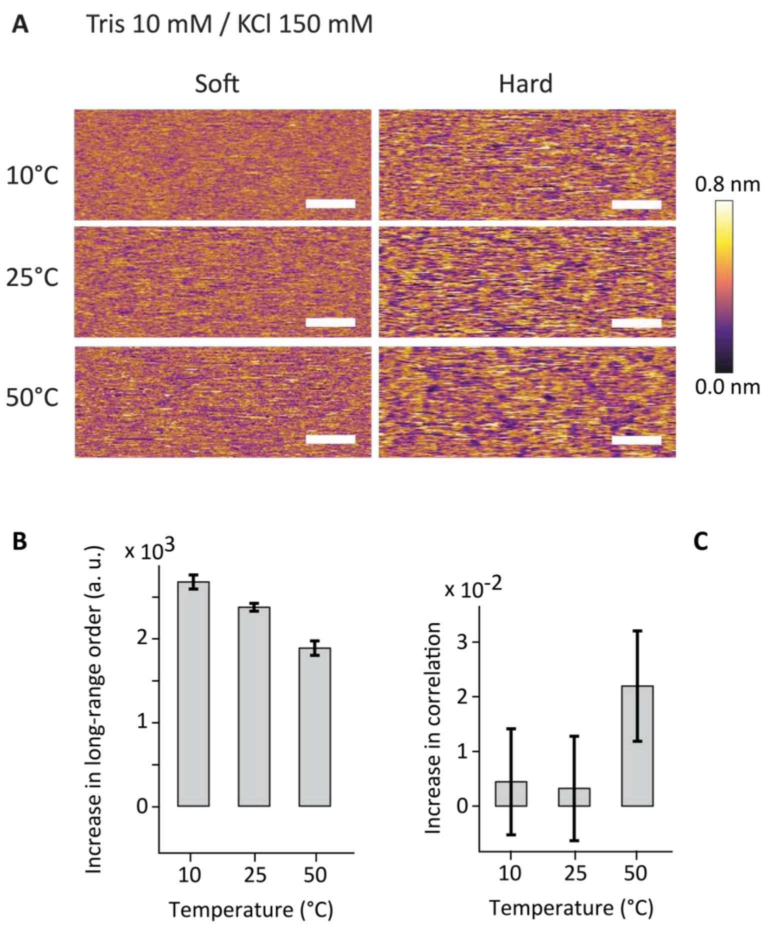

FIGURE S11. Effect of temperature on the stress response of DOPS membranes immersed in solution 4 (Tris 10 mM / KCl 150 mM). The topographic images shown no obvious evolution with temperature although nano-texture tends to be more marked at higher temperatures under Hard conditions. Quantitative analysis confirms this observations with correlation measurements (C) showing some increase at higher temperatures but no clear evolution within error. The increase in long-range order deduced from Fourier analysis (B) shows some decrease with increasing temperature, confirming that DOPS does not react as DOPC and tends to remain fluid throughout the experiment. The images scale bar is 50 nm.

12. COMPARISON OF TEXTURE FORMATION ON DIFFERENT LIPIDS (DOPC AND POPC)

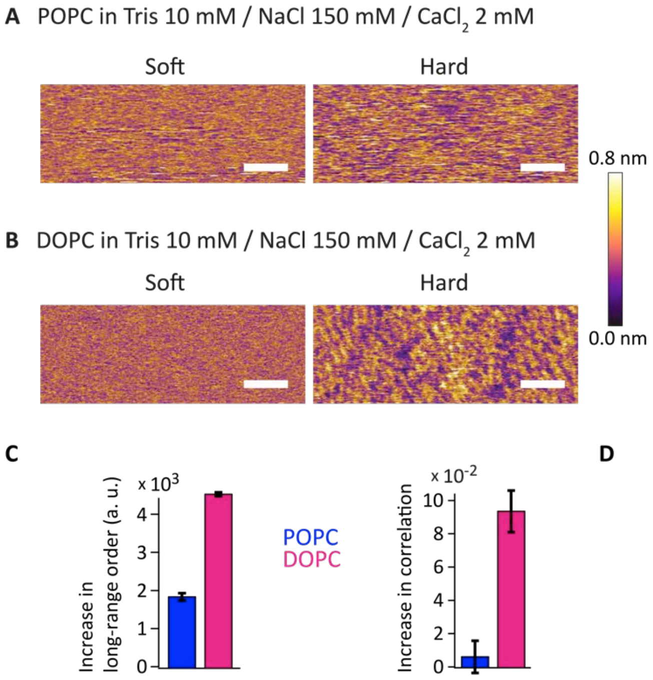

FIGURE S12. Comparison of the stress response of lipid bilayers composed of DOPC (1,2-dioleoyl-*sn*-glycero-3-phosphocholine) and POCP (1-palmitoyl-2-oleoyl-*sn*-glycero-3-phosphocholine) immersed in solution 7 (Tris 10 mM / NaCl 150 mM / $CaCl_2$ 2 mM). The topographic images indicate little change in POPC under stress (A) but significant changes in DOPC under the same conditions (B). This is confirmed by Fourier (C) and correlation (D) analysis of the change between Soft and Hard imaging conditions. The scale bar is 50 nm in (A) and (B).

13. SUPPORTING REFERENCES

1. Mingeot-Leclercq, M. P., M. Deleu, R. Brasseur, and Y. F. Dufrene. 2008. Atomic force microscopy of supported lipid bilayers. *Nat. Protoc.* 3:1654-1659.
2. Blumenthal, D., L. Goldstien, M. Edidin, and L. A. Gheber. 2015. Universal Approach to FRAP Analysis of Arbitrary Bleaching Patterns. *Sci. Rep.* 5:11655.
3. Pincet, F., V. Adrien, R. Yang, J. Delacotte, J. E. Rothman, W. Urbach, and D. Tareste. 2016. FRAP to Characterize Molecular Diffusion and Interaction in Various Membrane Environments. *Plos One.* 11:e0158457.
4. Veatch, S. L., and S. L. Keller. 2003. Separation of liquid phases in giant vesicles of ternary mixtures of phospholipids and cholesterol. *Biophys. J.* 85:3074-3083.
5. Müller, D. J., M. Amrein, and A. Engel. 1997. Adsorption of biological molecules to a solid support for scanning probe microscopy. *J. Struct. Biol.* 119:172-188.
6. Veatch, S. L., and S. L. Keller. 2002. Organization in lipid membranes containing cholesterol. *Phys. Rev. Lett.* 89:268101.
7. Stevens, M. M., A. R. Honerkamp-Smith, and S. L. Keller. 2010. Solubility Limits of Cholesterol, Lanosterol, Ergosterol, Stigmasterol, and beta-Sitosterol in Electroformed Lipid Vesicles. *Soft Matter.* 6:5882-5890.
8. Ma, Y., S. K. Ghosh, D. A. DiLena, S. Bera, L. B. Lurio, A. N. Parikh, and S. K. Sinha. 2016. Cholesterol Partition and Condensing Effect in Phase-Separated Ternary Mixture Lipid Multilayers. *Biophys. J.* 10:1355-1366.
9. Almeida, P. F. 2011. A simple thermodynamic model of the liquid-ordered state and the interactions between phospholipids and cholesterol. *Biophys. J.* 100:420-429.
10. Gumi-Audenis, B., L. Costa, F. Carla, F. Comin, F. Sanz, and M. I. Giannotti. 2016. Structure and Nanomechanics of Model Membranes by Atomic Force Microscopy and Spectroscopy: Insights into the Role of Cholesterol and Sphingolipids. *Membranes (Basel, Switz.).* 6.
