# Supplementary material for: Ions Modulate Stress-Induced Nanotexture in Supported Fluid Lipid Bilayers
Source: Biophys J. 2017 Jul 25;113(2):426–39. doi: 10.1016/j.bpj.2017.05.049 (PMC5529180; doi:10.1016/j.bpj.2017.05.049)
Supplement: Document S2. Article plus Supporting Material [file mmc2.pdf]

Biophysical Journal
Article

Biophysical Society

Ions Modulate Stress-Induced Nanotexture in Supported Fluid Lipid Bilayers

Luca Piantanida,[1] Hannah L. Bolt,[2] Neshat Rozatian,[2] Steven L. Cobb,[2] and Kislon Voïtchovsky[1,*]
[1]Department of Physics and [2]Department of Chemistry, Durham University, Durham, United Kingdom

ABSTRACT Most plasma membranes comprise a large number of different molecules including lipids and proteins. In the standard fluid mosaic model, the membrane function is effected by proteins whereas lipids are largely passive and serve solely in the membrane cohesion. Here we show, using supported 1,2-dioleoyl-*sn*-glycero-3-phosphocholine lipid bilayers in different saline solutions, that ions can locally induce ordering of the lipid molecules within the otherwise fluid bilayer when the latter is supported. This nanoordering exhibits a characteristic length scale of ~20 nm, and manifests itself clearly when mechanical stress is applied to the membrane. Atomic force microscopy (AFM) measurements in aqueous solutions containing NaCl, KCl, $CaCl_2$, and Tris buffer show that the magnitude of the effect is strongly ion-specific, with Ca^{2+} and Tris, respectively, promoting and reducing stress-induced nanotexturing of the membrane. The AFM results are complemented by fluorescence recovery after photobleaching experiments, which reveal an inverse correlation between the tendency for molecular nanoordering and the diffusion coefficient within the bilayer. Control AFM experiments on other lipids and at different temperatures support the hypothesis that the nanotexturing is induced by reversible, localized gel-like solidification of the membrane. These results suggest that supported fluid phospholipid bilayers are not homogenous at the nanoscale, but specific ions are able to locally alter molecular organization and mobility, and spatially modulate the membrane's properties on a length scale of ~20 nm. To illustrate this point, AFM was used to follow the adsorption of the membrane-penetrating antimicrobial peptide Temporin L in different solutions. The results confirm that the peptides do not absorb randomly, but follow the ion-induced spatial modulation of the membrane. Our results suggest that ionic effects have a significant impact for passively modulating the local properties of biological membranes, when in contact with a support such as the cytoskeleton.

INTRODUCTION

The plasma membrane separates the inside of the cell from the surrounding environment. It is a highly organized and heterogeneous structure composed of phospholipids, proteins, and organic molecules (1), and plays a fundamental and active role in the cell function. The lipids, which are the main constituent of the membrane, form a fluid double layer that embeds and anchors active biomolecules. However, the interplay between lipids and host molecules in the membrane is still poorly understood. The first widely accepted membrane model (2), the so-called "fluid mosaic model", assumes that phospholipids are merely structural molecules in which membrane proteins can move freely. This passive description of the membrane lipids is increasingly challenged by recent findings (3–6). Studies have shown that the membrane is a highly dynamical structure that actively supports the cell function. Phospholipids play an important role in this new understanding, for example in conferring membrane curvature (7) and actively organizing proteins complexes (6,8). Despite these advances, our molecular-level understanding of plasma membranes is still limited, partly due to a lack of experimental results. Important questions related to the anomalous diffusion of lipids under certain circumstances (9,10) or the role of membrane rafts (11) remain a matter of debate. Molecular-level studies of natural biomembranes are usually challenging due to the large number of different molecules in the membrane as well as their specific organization. To overcome these difficulties, many studies make use of synthetic lipid bilayers as model biological membranes. The advantage of synthetic bilayers is the possibility of controlling the membrane composition precisely, therefore helping the interpretation of experiments while retaining some of the most important features of natural biomembranes (12–15). Additionally, when addressing single-molecule details, it is often helpful to support the membrane on a substrate. In cells, free-standing plasma membranes constantly reshape (16) and undulate (17) to help cellular function, rendering

Submitted December 6, 2016, and accepted for publication May 30, 2017.
*Correspondence: kislon.voitchovsky@durham.ac.uk
Editor: Stephen Evans.
http://dx.doi.org/10.1016/j.bpj.2017.05.049
© 2017 Biophysical Society.
This is an open access article under the CC BY license (http://creativecommons.org/licenses/by/4.0/).

CrossMark

local tracking of molecules challenging. Solid-supported membranes are typically immersed in aqueous solution, and only a few nanometers of the solution separate the membrane from the solid support (18). This approach effectively confines the membrane in two dimensions, hence facilitating high-resolution investigations with techniques such as atomic force microscopy (19), fluorescence microscopy (20), fluorescence recovery after photobleaching (FRAP) (21), quartz crystal microbalance (22), and neutron reflectometry (6). Synthetic supported lipid bilayers (23) (SLBs) are routinely used in experimental studies, including for investigating the influence of proteins (24,25), antibodies (26), functionalized nanoparticles (27), polymers (28), amino acids (29), and antimicrobial peptides (30) on the morphology and biophysical properties of the membrane.

The presence of a supporting solid does, however, affect the behavior of SLBs. Simple biophysical properties such as the lipids transition temperature (31) and molecular diffusion coefficients have been shown to differ in SLBs when compared with identical membranes free-standing in solution (23). This can be partly explained by interactions between the lipids of SLBs and the substrate. These interactions can be direct such as van der Waals and electrostatic forces (12,32), or indirect, through a global hydrogen bond network that stabilizes the membrane when immersed in a solution. The hydrophilicity of the substrate can affect the thickness and the dynamics of the water trapped between the substrate and the bilayer (33). Not surprisingly, the formation and properties of SLBs can be tuned by changes in the lipid-substrate interactions, for example, through the solution's pH (34), ionic strength (35), electrolyte content (36–38), by temperature (39), and the substrate's topography and chemistry (40). The fact that SLBs' properties differ from those of free-standing bilayers is often seen as a limitation of SLB-based experiments, with natural biomembranes expected to behave more like freestanding bilayers. However, natural cell membranes are never completely free-standing, but locally supported and constrained by the cytoskeleton and cytoplasm on one side, and the extracellular matrix on the other side. Local interactions between these supports and the molecules composing the membranes are believed to play an active role in the membrane function and to be key to the cell survival (41). The interaction of SLBs with the substrate therefore represents an opportunity to examine the influence of cell support on the local properties of the membrane. Given the extended and homogenous SLB-substrate interaction in most experiments, it is necessary to investigate the membrane locally, and at the nanoscale, if results are to be relevant for our understanding of natural systems.

Atomic force microscopy (42) (AFM) is a tool of choice to conduct this type of experimental study; it operates locally at the nanoscale (43), can function in the liquid environment (44), and can quantify not only topography (45) but also the nanomechanical and viscoelastic properties (46) of soft biological samples. Studies of SLBs with AFM in the liquid environment (19,47) have allowed identification of nanoscale features such as different lipid microdomains (48), and submolecular details of membrane protein structure (49) and function (50). When operated dynamically (vibrating tip) and with small tip oscillation amplitudes, it is also possible to derive molecular-level information about the membrane hydration landscape (51) including in the presence of adsorbed ions (52,53) that can often be directly imaged (54,55). Recent AFM studies have shown that ions can form correlated clusters at the surface of mica in solution (56). Ions have long been known to interact with biomembranes (37,57) and sometimes specifically with lipids (58). Metal cations can bind to different locations of the lipid headgroups of common plasma membrane's lipids such as phosphatidylcholine or phosphatidylserine (38,59,60). This often results in significant changes of the membrane's characteristics (61,62). However, the effect of ions on the substrate-bilayer interaction is still poorly understood at the mesoscale—the 1–100 nm range—where correlation and clustering effects are expected to dominate (63). In that range, a small number of molecules acting in a coherent manner can induce local but significant changes in the membrane's properties. Furthermore, the role of macroions such as commonly used buffer molecules is generally ignored. Buffers and metal ions trapped between the substrate and the bilayer can significantly alter the hydration landscape of the proximal leaflet, potentially affecting the local nanomechanics and dynamics of the supported lipid bilayer surface.

In this study we use AFM to investigate the effect of different metal and buffer ions on the interaction between fluid phospholipid SLBs with the underlying substrate in solution. We focus on the nanoscale organization of the lipids when under local confinement. We show that the average pressure applied by the AFM tip while imaging the SLB could trigger the formation of long-lived but reversible mesoscale structures, likely due to a localized gelation-like ordering of lipid molecules in the membrane. This phenomenon depends strongly on the ionic species present in the solution used for creating the SLB. We correlate the AFM results with the average lateral diffusion coefficient of the lipids in the membrane, obtained from FRAP measurements. The results are discussed from the perspective of nanoscale lipid mobility and molecular interactions. We also discuss the importance of ion-induced mesoscale effects for modulating the adsorption of biomolecules, illustrated here with the cell-penetrating antimicrobial peptide Temporin L.

MATERIALS AND METHODS

Materials

All chemicals were obtained from commercial sources and used without further purification. The lipids, 1,2-dioleoyl-*sn*-glycero-3-phosphocholine

(DOPC), 1-palmitoyl-2-oleoyl-*sn*-glycero-3-phosphocholine (POPC), and 1,2-dioleoyl-*sn*-glycero-3-phospho-L-serine (DOPS), where purchased, were dissolved in chloroform from Avanti Polar Lipids (Alabaster, AL). 1,2-dipalmitoyl-*sn*-glycero-3-phosphoethanolamine-N-(lissamine rhodamine B sulfonyl, ammonium salt) (DPPE-Rhod) in powder form was obtained from the same company. The salts and buffering agents (all >99% purity) were purchased from Sigma-Aldrich (Dorset, UK) and dissolved/diluted in ultrapure water (Merck-Millipore, Watford, UK). Solutions were created with fixed concentrations of ions as follows: KCl 150 mM, NaCl 150 mM, Tris 10 mM, $CaCl_2$ 2 mM, combining them in the eight different solutions listed in Table 1. Wherever possible, the pH was adjusted to 7.4 using HCl 5 M and KOH/NaOH 5 M (depending on whether NaCl or KCl was used in the solution).

SLB preparation

SLBs of DOPC, POPC, and DOPS were formed via the vesicle fusion method (13,64). In short, 100 μL of a chloroform solution containing the lipids (10 mg/mL for DOPC and POPC, and 1 mg/mL for DOPS) was pipetted into a 3 mL glass vial and dried under a gentle nitrogen steam until no fluid remained. For FRAP experiments, 1.6 μL of DPPE-Rhod 0.4 mM (0.05% final concentration) was added to the DOPC before drying. The vial was then placed under vacuum for >12 h to eliminate residual chloroform. The resulting lipid film was subsequently rehydrated in 1 mL of the solution of interest so as to achieve a lipid concentration of 1 mg/mL. The vial was bath-sonicated for at least 30 min at >40°C until the solution became uniformly milky and opaque due to the formation of multilamellar vesicles. The solution was then extruded 11 times using a Mini-Extruder kit (Avanti Polar Lipids) with a Whatman 100 nm filter (GE Healthcare Life Sciences, Little Chalfont, UK) to form large unilamellar vesicles. The solution was further diluted 10 times in the same solution (except DOPS), to reach a final concentration of 0.1 mg/mL. A drop (100 μL) of this solution was deposited on a disk of Grade I freshly cleaved Muscovite mica (SPI Supplies, West Chester, PA). The sample was incubated 30 min at 65°C in a sealed Petri dish and then progressively cooled down to 25°C over 1.5 h to ensure full relaxation of the bilayer in its fluid state. The incubation was done in a water-saturated atmosphere to prevent any drying of the sample. The sample was then rinsed carefully with measurement solution (15× diluted preparation solution) before AFM imaging. This last procedure serves two purposes: first, because this study focuses on the influence of the substrate on the bilayer's behavior, removal of some of the ions in solution emphasizes the relative importance of the ions trapped between the bilayer and the substrate in modulating SLB-substrate interactions. Second, the rinsing does not affect the already formed bilayer but it allows clearer images of the surface's changes and minimizes tip-induced effects (65) (for details, see Fig. S1). Because the DOPC bilayer is in fluid phase at the experimental temperature (25°C), any exposure of the sample to air (bubbles or drying) disrupts the bilayer assembly. It was therefore not possible to use the same bilayer for all the experiments (with solution exchange) and new samples were prepared for each experiment. All the glassware, and utensils used in the procedure were first thoroughly cleaned by sonication in, sequentially, an aqueous solution of Decon-90 detergent (Deacon Laboratories, Brighton, UK), ultrapure water, 20% isopropyl alcohol, and ultrapure water again, each time for 10 min.

TABLE 1 Detailed Composition of the Eight Different Deposition Solutions Used for the Formation of SLBs

Solution Reference Number	Solution Composition
1	KCl 150 mM
2	NaCl 150 mM
3	Tris 10 mM/NaCl 150 mM
4	Tris 10 mM/KCl 150 mM
5	NaCl 150 mM/$CaCl_2$ 2 mM
6	KCl 150 mM/$CaCl_2$ 2 mM
7	Tris 10 mM/NaCl 150 mM/$CaCl_2$ 2 mM
8	Tris 10 mM/KCl 150 mM/$CaCl_2$ 2 mM

The measurement solutions were obtained in each case by diluting the deposition solutions with ultrapure water 15 times.

The antimicrobial peptide Temporin L (see below for details on the synthesis) was first equilibrated in Tris 10 mM/NaCl 150 mM, pH 7.4 ± 0.2 stock solution at a concentration of 1.3 mM. Having the peptide solution already buffered at neutral pH avoids the pH-induced changes in the conformation of the peptide. In the comparative AFM analysis, 30 μL of the stock solution were added to the sample (100 μL), obtaining a final concentration of ~0.3 mM. When used in solution 6, the initial pH of the solution (6.0 ± 0.5) was brought near neutral value (pH 7.0 ± 0.2) by the adjunction of the peptide solution, allowing direct comparison between the different solutions without concerns for pH effects on the peptide structure. Solution 7 is already buffered and no measurable pH change was observed upon adjunction of Temporin (pH 7.4 ± 0.2).

Membrane penetrating peptide Temporin L synthesis

Peptide synthesis grade DMF was obtained from AGTC Bioproducts (Hessle, UK), PyBOP from Apollo Scientific (Stockport, UK) and all resins and amino acids were purchased from Novabiochem by Merck (Darmstadt, Germany). These chemicals were used without further purification and stored under appropriate conditions, as detailed in the manufacturer's instructions. Bond Elut solid phase extraction cartridges (20 mL, polypropylene with two polypropylene frits) were purchased from Crawford Scientific (Strathaven, UK) and used as reaction vessels for solid phase synthesis. Solvents were removed under reduced pressure using a Büchi Rotavapor R1 (Flawil, Switzerland), a shaker was used to mix solutions during solid phase synthesis, and aqueous solutions were lyophilized using a Christ Alpha 1-2 LD Plus (Osterode am Harz, Germany) freeze-drier.

Temporin L (sequence FVQWFSKFLGRIL-NH_2) was synthesized via manual solid phase peptide synthesis. Amino-acid side-chain functionality was protected as follows: Fmoc-Arg(Pbf)-OH, Fmoc-Gln(Trt)-OH, Fmoc-Lys(Boc)-OH, Fmoc-Ser(tBu)-OH, and Fmoc-Trp(Boc)-OH. Fmoc-protected Rink Amide resin (0.1 mmol, 128 mg, loading 0.78 mmol g^{-1}) was swollen in DMF overnight at room temperature in a 20 mL polypropylene syringe fitted with two polyethylene frits. The resin was deprotected with piperidine (20% in DMF v/v, 2 × 20 min) and washed with DMF (3 × 2 mL). PyBOP (4 equivalents with respect to the resin), the Fmoc-protected amino acid (4 equivalents), and DIPEA (4 equivalents) were dissolved in the minimum DMF and left to preactivate for several minutes, before being added to the resin and placed on the shaker at 400 rpm at room temperature for 1 h. The resin was then washed with DMF (3 × 2 mL) and deprotected using piperidine (20% in DMF v/v, 2 × 20 min) and washed as before. Further amino acid couplings and Fmoc-deprotection steps were made as necessary to complete the full sequence. The resin was washed with DMF (3 × 2 mL) and then DCM (3 × 2 mL) to remove DMF before resin cleavage. Final cleavage from the resin was achieved using 95:2.5:2.5 TFA/H_2O/TIPS (4 mL). The resin was then placed on the shaker at 400 rpm for 4 h and the resin removed by filtration. The cleavage cocktail was removed in vacuo, the crude product precipitated in diethyl ether (45 mL) and the precipitate retrieved by centrifuge for 15 min at 5000 rpm. The ether phase was decanted, the crude product dissolved in a mixture of acidified H_2O and MeCN and lyophilized before RP-HPLC purification. The crude peptide was dissolved into ~1.5 mL (95% H_2O, 5% MeCN, 0.1% TFA) and purified by preparative RP-HPLC using a 200 Series LC pump (PerkinElmer, Wokingham, UK) with a 785A UV-vis detector ($\lambda = 220$ nm; PerkinElmer) on an SB Analytical column (ODS-H Optimal; Crawford Scientific), 250 × 10 mm, 5 μm; flow rate = 2 mL min^{-1}; and typical linear gradient elution 0–100% solvent B over 80 min (solvent A = 0.1% TFA in 95% H_2O, 5% MeCN; solvent B = 0.1% TFA in 5% H_2O, 95% MeCN). Relevant fractions were collected, lyophilized, and analyzed by LC-MS and analytical RP-HPLC to yield

Temporin L as a white powder (38 mg, 23%); RP-analytical HPLC RT 17.8 min, approximate purity >99%, and QToF MS mass calculated *m/z* $[M+H]^+$ 821.0, with mass observed $[M+2H]^{2+}$ 820.7. The characterization data described can be found in Fig. S2.

AFM measurements

A commercial AFM (Cypher ES; Asylum Research, Santa Barbara, CA) equipped with direct laser excitation (blueDrive; Asylum Research) and temperature control was used for all the experiments. The blueDrive and temperature control enable ultrastable operation and fully reproducible imaging parameters. This is important for allowing direct comparison between results acquired in the different imaging solutions. All measurements, except for the controls presented in Fig. S3, were conducted using cantilevers from the same wafer for better comparability (OMCL RC800-PSA; Olympus, Tokyo, Japan). The cantilevers have a nominal spring constant of 0.39 N/m and systematic thermal calibration (44,66) showed variations of <10% between cantilevers. The tip is pyramidal shape with a curvature radius ≤15 nm at its apex. Imaging was carried out in amplitude modulation with the cantilever driven at its fundamental resonance frequency (~25 kHz in liquid) for all the experiments. In amplitude modulation mode, the cantilever is oscillated near resonance frequency and the amplitude is kept constant while scanning. The optical level sensitivity was calibrated before the experiments. The free amplitude A_{free} away from the interface was set to $A_{\mathrm{free}} = 2.0$ nm. Images were acquired at setpoint amplitudes A_{set} of 1.6, 1.2, and 0.6 nm for each sample, corresponding, respectively, to setpoint ratio $A_{\mathrm{set}}/A_{\mathrm{free}}$ of 0.8 ("Soft" imaging conditions), 0.6 ("Medium" imaging conditions), and 0.3 ("Hard" imaging conditions). In all cases, it was ensured that the tip did not traverse the bilayer while oscillating. If such an event occurs, it can be immediately identified by dramatic changes in the resulting image due to the tip tapping the hard substrate underneath the SLB. For the control experiments shown in Fig. S3, a cantilever carrying a rounded tip (radius of curvature of ~30 nm) made of the same material as the main tips was used (R30 FM; Nanosensors, Neuchatel, Switzerland).

Analysis of the AFM images

All the images were corrected for tilt (line or plane flattening) and lightly low-pass filtered to remove grainy noise using the WSxM software (Nanotec Electronica, Madrid, Spain) (67). Depending on the imaging solution, the membrane progressively became gel-like when decreasing the setpoint ratio (i.e., turning to Hard imaging conditions). This transition induces the apparition of stable nanofeatures or nanotexturing of the membrane that remains in place throughout the imaging process. Quantification of the apparent surface structure was done with homemade routines programmed in the software Igor Pro (WaveMetrics, Lake Oswego, OR). Because objectively quantifying the degree of order or the presence of stable nanostructures is challenging, two different criteria were used to evaluate long-range and short-range lateral order, respectively.

The first method, based on Fourier analysis, was used to quantify the relative increase on long-range (>150 nm) order in the images, averaged over all directions. Long-range order is expected to increase significantly if the membrane undergoes a transition from fluid to gel-like. Here the analysis is done by examining the 2D fast Fourier transforms (2D FFT) of height images. A profile is averaged over all directions and back-transformed into real space (see Fig. S4, *A–C*). The resulting curves (one per topographic image, Fig. S4 *C*) exhibit local maxima where features of a given size are more abundant in the original image. The change C_{FFT} in long-range order is then calculated using the following:

$$C_{\mathrm{FFT}} = \left| \int_{s1}^{s2} P_{\mathrm{FFT}}^{\mathrm{Hard}}(s) - P_{\mathrm{FFT}}^{\mathrm{Soft}}(s)\mathrm{d}s \right|, \tag{1}$$

where $P_{\mathrm{FFT}}^{\mathrm{Hard}}$ and $P_{\mathrm{FFT}}^{\mathrm{Soft}}$ are the averaged profiles for the images acquired in Hard and Soft conditions, respectively, and $s1$, $s2$ values are the interval in feature sizes investigated. In $P_{\mathrm{FFT}}^{\mathrm{Hard}}$, local maxima are clearly visible for feature sizes that are multiples of ~20 nm, the characteristic size of stress-induced gel-like protuberances. However, even when little stress is applied to the membrane (Soft and Medium conditions), some local maxima can be seen in the 10–50-nm size interval due to tip-induced fluctuations of the fluid membrane. The best indicator of membrane localized gelation is hence the existence of long-range order, obtained by taking [150; 300 μm] for the [$s1$;$s2$] interval. C_{FFT} is a quantitative indicator of the change in long-range order in a given sequence of images. However, its absolute value does not contain physically meaningful information and C_{FFT} can hence be normalized to the largest value when comparing different sequences (as in Fig. 3).

The second method analyses the average correlation between adjacent linescans. It complements the Fourier analysis by quantifying the existence of short-range order, taking into account tip-induced fluctuations of a fluid membrane. If two adjacent profiles, as scanned by the tip, exhibit a high degree of similarity, then the resulting correlation will be close to unity (maximum value), regardless of the shape of the profile. Fluctuations over fluid membranes are hence likely to decrease the correlation (Fig. S4 *D*). Let us consider a height profile line $P_{\mathrm{Corr}}^{\mathrm{line}\ n}(x)$ obtained from the raster scan line number n of the AFM tip over the image. The parameter x designates a point along the profile. The image being 256 × 256 pixels, we have $0 \leq n \leq 255$. We define $max_{\mathrm{line}\ n}$, $min_{\mathrm{line}\ n}$, and $avg_{\mathrm{line}\ n}$ as the numerical values of the maximum height, minimum height, and average height of $P_{\mathrm{Corr}}^{\mathrm{line}\ n}$. We can then define a normalized profile $NP_{\mathrm{Corr}}^{\mathrm{line}\ n}$ as follows:

$$NP_{\mathrm{Corr}}^{\mathrm{line}\ n} = \frac{P_{\mathrm{Corr}}^{\mathrm{line}\ n} - avg_{\mathrm{line}\ n}}{max_{\mathrm{line}\ n} - min_{\mathrm{line}\ n}}. \tag{2}$$

This normalization procedure ensures that each $NP_{\mathrm{Corr}}^{\mathrm{line}\ n}$ is centered on zero (no vertical offset) and has a maximum height variation of 1. We can then calculate the degree of correlation $Corr_{n,\ n+1}$ between the two adjacent lines n and $n+1$, as follows:

$$Corr_{n,\ n+1} = 1 - \frac{\int_x \left| NP_{\mathrm{Corr}}^{\mathrm{line}\ n}(x) - NP_{\mathrm{Corr}}^{\mathrm{line}\ n+1}(x) \right| \mathrm{d}x}{\int_x \left| NP_{\mathrm{Corr}}^{\mathrm{line}\ n}(x) \right| + \left| NP_{\mathrm{Corr}}^{\mathrm{line}\ n+1}(x) \right| \mathrm{d}x}, \tag{3}$$

where the integral is calculated over the whole length of the profile. Defined in this manner, we have $0 \leq Corr_{n,\ n+1} \leq 1$, with $Corr_{n,\ n+1}$ corresponding to a situation where the two profiles are identical and the values close to zero to a low degree of correlation. We note that other definitions of correlation are possible, but this definition has the advantage of being intrinsically normalized, with a simple interpretation of the values obtained. We can then calculate a single correlation value $Corr$ for any given image by averaging all the correlation results $Corr_{n,\ n+1}$ for $0 \leq n \leq 255$. The uncertainty is taken as the standard error.

Quantitative analysis of the image features was conducted on the raw data without any prior processing except for tilt correction.

FRAP measurements

FRAP measurements were conducted on the DOPC SLBs to quantify lipid mobility in each solution. As for AFM, the FRAP measurements were conducted after rinsing of the SLBs with a diluted solution. A fluorescently labeled lipid (DPPE-Rhod; Avanti Lipids) was added to the DOPC bilayer (0.05%). Its low concentration does not affect the overall DOPC fluidity but the Rhodamine tag provides the fluorescence needed. After the SLB formation, the sample was placed in an EZ-C1 Confocal Microscope (Nikon UK, Kingston, UK) and imaged in reflection mode. The fluorescence recovery was analyzed over a 10 × 10 μm^2 bleaching spot within a 500 × 500 μm^2 image. We acquired 45 frames (1 s/frame, 2 s gap between consecutive frames) over a total duration of 1 min and 30 s. At least three

frame sequences were acquired for each sample so as to optimize the measurement parameters, ensure reliability of the result, and derive statistically meaningful results. The dark background was avoided to prevent modification of the digital contrast. In cases where the bilayer did not cover the whole sample uniformly, we centered the bleaching spot at the center of the largest-as-possible patches (see also Fig. S5 for details).

The images sequences obtained from the FRAP measurements were processed with a user-defined macro available in the software ImageJ (National Institutes of Health, Bethesda, MD). The resulting time-dependent fluorescence intensity data were processed with in-built analysis tools in the software Igor Pro (WaveMetrics). The geometry of the bleaching spot being squared, the standard fitting equations (68) normally used to derive the diffusion coefficient (D) were adapted based on the derivation given in reference (69) and used to fit time-dependent intensity data, as follows:

$$I(t) = a_0 + a_1\left(1 - \sqrt{\frac{w^2}{w^2 + 4\pi D(t - t_{\text{bleach}})}}\right), \quad (4)$$

where w (in micrometers) is the width of the square spot area and D (square micrometer per second) is the diffusion coefficient of the lipid molecules in the bilayer (see Fig. S5). The diffusion coefficients derived in each case were averaged over the different sequences captured and the stated uncertainty is the standard error of the average. Higher uncertainties values are found in presence of smaller SLB patches due to edge effects (see Supporting Material discussion accompanying Fig. S5).

RESULTS

Mechanical stress induces ion-dependent nanoscale texturing of the bilayer

Fig. 1 shows representative AFM images of DOPC SLBs formed in the eight different saline solutions used. In each case, images acquired in scanning conditions described as Soft, Medium, and Hard are given, corresponding to setpoint ratios of 0.8, 0.6, and 0.3, respectively (see Materials and Methods for details).

When imaged in Soft conditions, the average pressure exerted by the tip on the membrane is minimal and no particular features are visible, except for occasional protrusions due to unfused vesicles. The membrane is fluid and the tip tends to sweep molecules as it scans across the surface, leading to relatively noisy images characteristic of AFM operation over fluid bilayers. As the imaging conditions progressively change from Soft to Hard, the average pressure exerted by the tip on the membrane increases and the appearance of the membrane can change dramatically, depending on the ionic content of the solution used for the bilayer formation. To emphasize the impact of ions in contact with the proximal leaflet (where the bilayer is supported), the SLB was rinsed with a diluted ionic solution (measurement solution) immediately before imaging (see Fig. S1). Using solutions containing only monovalent ions or divalent ions with Tris buffer, little change is visible, as exemplified by solution 7 (Tris 10 mM/NaCl 150 mM/$CaCl_2$ 2 mM). In contrast, in solutions containing no Tris and divalent ions, clear nanoscale structures start to emerge. This is particularly visible in solution 6 (KCl 150 mM/$CaCl_2$ 2 mM) where the bilayer surface orders itself in a corrugated profile composed of an extended network of 10–20-nm-wide and ~0.8-nm-deep valleys and ridges spanning the imaged area. These stable peaks and valleys create a nanoscale pattern hereafter referred to as a "nanotexture" for simplicity. These structures appear stable while the tip pressure is maintained and consecutive images acquired in Hard conditions show reproducible nanoscale features, despite DOPC being normally fluid at the experimental temperature of 25°C (transition temperature $T_m = -17°C$ in solution). This gel-like nanotexture appears almost instantaneously when pressure is applied by the scanning tip, pointing to a tip-induced effect. The stability of this nanotexture is reminiscent of a fluid-to-gel-phase transition, hence our use of "gel-like", but these results do not characterize the nature of this transition. The texture eventually disappears if the tip is moved away from the surface of the membrane. This can be seen when changing scan conditions between consecutive images, suggesting a stress relaxation occurring over minutes (see Fig. S6). There is an apparent opposing action of Tris and $CaCl_2$, both working in conjunction with the monovalent salts. When both species are present in the solution the fate of the membrane under stress becomes difficult to predict. Some subtle differences can be seen between the monovalent ions. There is, for example, a clearer texture definition in solution 3 than in solution 4, but between solutions 5 and 6, the situation is reversed. Adjunction of cholesterol in the membrane only reinforces this effect (Fig. S7). This is hardly surprising given the different physico-chemical properties of cholesterol, but the result suggests that stress-induced nanotexturing may be a common feature of biological membranes.

Determining the origin of these nanostructures is not straightforward because the measurement process plays a role in their formation. First, interactions between the imaging tip and the lipid bilayer can influence both the membrane and the observations. Here, to limit tip-induced disruption of the membrane, the AFM is operated in amplitude modulation, with the tip intermittently pressing the surface of the lipids. In contact mode, a similar nanotexture of the DOPC SLBs could be observed (Fig. S8 for an example in solution 6). However, the shearing motion of the tip in contact with the membrane renders this mode of imaging unstable and the tip tends to traverse the membrane even when using ultrasoft imaging conditions. In amplitude modulation, the imaging is fully stable. Depending on the imaging conditions (Soft, Medium, or Hard) the tip probes the hydration landscape at the surface of the membrane or the nanomechanical properties of the membrane itself (70,71). In practice, this is quantitatively controlled by the imaging setpoint (see Material and Methods) that can be almost instantaneously readjusted at any time. In Hard imaging conditions, the setpoint is relatively low (~30% of the free amplitude) and the tip transiently presses on the lipid surface during every oscillation cycle. Because the fast tip

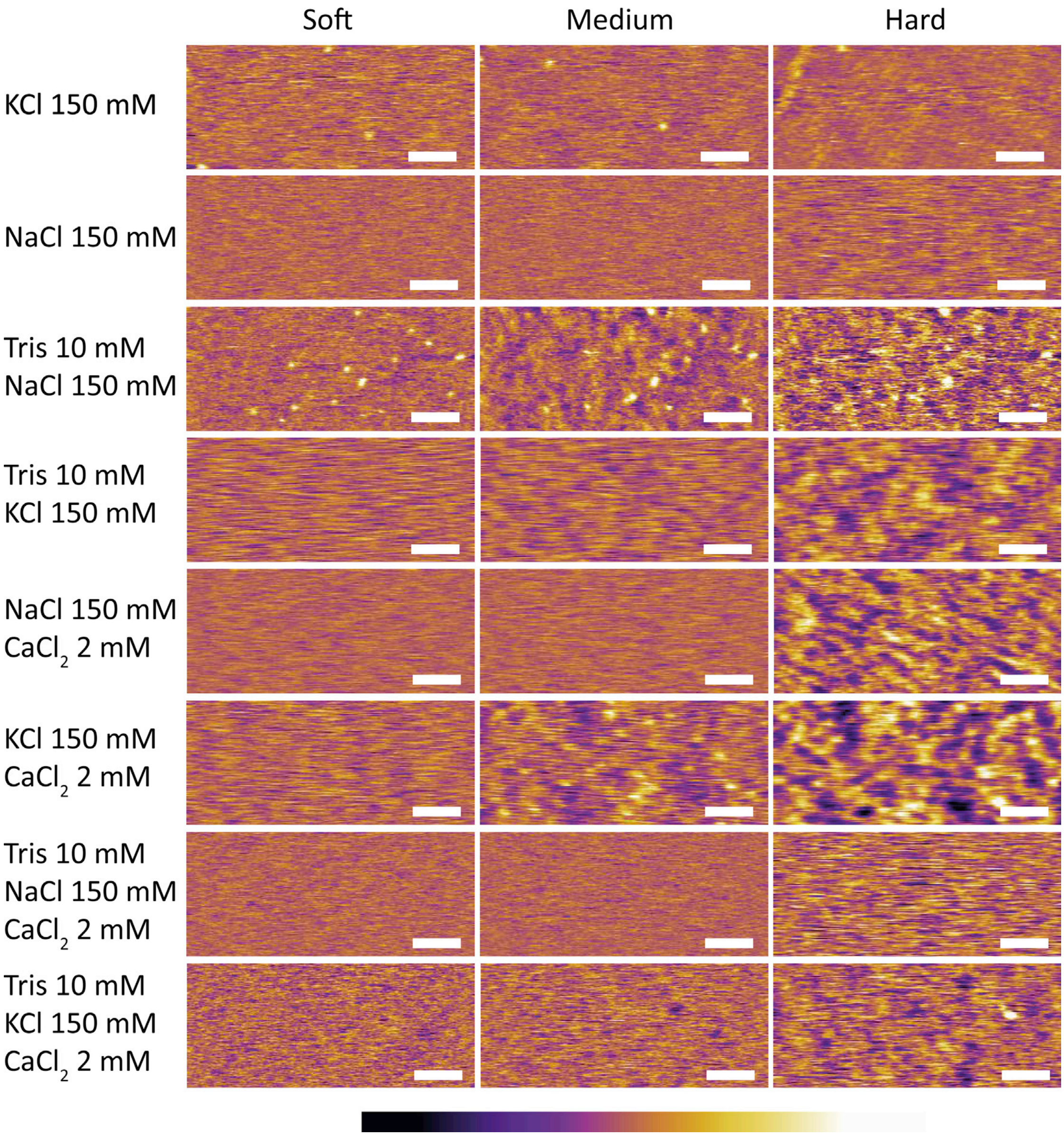

FIGURE 1 Comparative analysis of representative AFM micrographs obtained on DOPC SLBs in each imaging solution. The average force exerted by the imaging tip on the SLB gradually increases as the scanning conditions change from Soft to Medium to Hard (corresponding, respectively, to setpoint values of 0.8, 0.6, and 0.3). The images acquired in solution 7 (Tris 10 mM/NaCl 150 mM/$CaCl_2$ 2 mM) exhibit little influence of the scanning conditions on the appearance of the SLB's surface. In contrast, images acquired in solution 6 (KCl 150 mM/$CaCl_2$ 2 mM), show an obvious increase in surface roughness and the apparition of clear topographic nanotexture as the setpoint decreases. Scale bars, 50 nm; color scale bars, height variation of 0.8 nm. To see this figure in color, go online.

motion is vertical with respect to the lipid surface, no significant shear is imposed. The nanoscale structures therefore appear to be induced by normal compression of the membrane, but the tip only dwells a few microseconds in the same location before moving on.

Quantifying the maximum force F and the average pressure P locally applied by the tip to the SLB is challenging. The tip oscillates at the resonance frequency of the cantilever, and its instantaneous deflection is not directly related to the applied force (72,73). Here, we are only interested in estimating the orders of magnitude of F and P and therefore use the following approximation: $F \sim kA_0(1 - S)$, where k is the spring constant of the cantilever, A_0 is the free amplitude, and S is the setpoint ratio. This approximation yields $F \sim 0.6$ nN in Hard imaging conditions, so a force is on the order of 0.1–1 nN, consistent with previous nanomechanical studies (73,74). For the standard AFM tip, such a force induces a tip indentation depth of typically less than a nanometer in a biomembrane (74,75). If we further assume that the contact area of the tip with the membrane is on

the order of 100 nm^2, the local tip-induced pressure in the membrane is on the order of $P \sim 1$–10 MPa. This pressure is comparable to the normal elasticity modulus (compressibility) of typical biomembranes (75,76), and can be naturally induced by proteins able to locally alter the molecular arrangement of the lipids (77). Control experiments conducted with a tip presenting a radius of curvature twice as large revealed a similar nanotexturing of the membrane, although with smaller height variations due to the tip's size. Quantitative analysis of the features sizes indicated no significant difference between results obtained with the two different tips, suggesting that the features are intrinsic to the DOPC SLB and not due to tip convolution effects (Fig. S3).

Second, the strong influence of the solution in which the SLB is formed on the apparition of nanotexture points to ion-modulated substrate-lipid interactions. The local organization of the lipid molecules is dependent on the composition of the solution, provided the membrane is under a stress stimulus. This effect is also present if the experiment is carried out in the formation solution (Fig. S1), but it appears more clearly in the diluted solution (Fig. 1). Ionic species present in solution are known to influence specifically the mechanical and diffusion properties of the lipid bilayers (35), but to the best of our knowledge, no results available to date have examined the impact of the solution between the bilayer and its support, in particular mesoscale effects. Here, the particular stress-induced surface texture imaged by AFM suggest the lipids behave similarly to shear-thickening liquids in rheology, with an effective viscosity determined by the ionic composition of the solution. The ions interact strongly enough with the lipids to trigger changes in their mobility within the membrane when under stress. Ion-mediated interactions between the lipids and the supporting substrate can, in principle, also stabilize the bilayer. It is known that several water layers are present between the lipids headgroups facing the mica (proximal leaflet) and the surface of the mica itself (32,70). Ions must also be present in this region given their key role in modulating the bilayer deposition from vesicles in solution (78), and buffer ions can form nanoscale features on mica (79). However, these ionic features appear on a scale typically smaller than that observed here (79) and the membrane is generally fluid (15). The effect of the substrate can hence be seen as global and mostly homogenous with regard to the membrane properties, but local interactions could explain the spatial reproducibility of the textures observed over the same area. To distinguish the effect of the ions on the proximal and the distal (exposed) leaflet, we conducted some experiments where the SLB was formed in ultrapure water, and the liquid subsequently gently exchanged with a KCl and/or Tris solution known to induce considerable nanotexture under AFM imaging. Such texture appeared under tip pressure only when ions were in the solution, and disappeared completely after subsequent thorough rinsing with pure water. In contrast, bilayers prepared by directly rehydrating the lipids in an ionic solution retained their ability to form textures even after several rinsing steps with water. This suggests a strong (almost irreversible) interaction between ions and the lipids, and confirms the limited influence of the substrate. To further support this finding, we have attempted to repeat our experiments on silicon oxide for its amorphous surface arrangement. The measurements were, however, inconclusive due to the intrinsic substrate's roughness, which is comparable to that of the stress-induced texture on the bilayer (Fig. S9).

Molecular origins of the stress-induced nanotexture

The results presented above demonstrate that the nanotexture induced by mechanical stress of the membrane is strongly dependent on the solution through ion-mediated substrate-lipid interactions. The results also show that the features composing this nanotexture are an intrinsic property of the DOPC SLB and do not significantly depend on the geometry of the AFM tip. Taken together, these findings hint at a local molecular rearrangement of the lipid when under stress so as to form solidlike nanonodules that return to the initial fluid bilayer arrangement once the applied stress is removed. In other words, the bilayer could undergo a local fluid to gel-like transition when under stress, a process influenced by the ions' ability to modulate substrate-lipid and lipid-lipid interactions. It is well known that bilayers can exhibit ripples or corrugations induced mechanically (80), electrically (81), or by temperature when in pretransition state (63,82–84). These effects depend on various parameters such as hydration, carbon chain length, and ion species that are present in solution (85–87). Here similar effects seem at play but locally, on the nanometer scale. Stable surface texture indicates a high degree of molecular ordering within the bilayer, comparable to a highly localized pretransition state. To verify this hypothesis, we conducted experiments similar to that presented in Fig. 1, but at different temperatures. The fluid-to-gel-phase transition temperature of SLBs has been shown to increase by up to 10°C compared to the same lipids in solution. The DOPC SLB remains fluid when imaged in Soft conditions, but if the AFM tip is able to induce local gelation-like molecular ordering, a shift of 15°C in the system temperature can be expected to have a significant effect. We have therefore explored the behavior of the SLB also at 10 and 50°C for a solution exhibiting limited stress-induced nanotexturing of the membrane (solution 7). The results, presented in Fig. 2, confirm that the tendency of the bilayer to form nanotexture increases at lower temperatures whereas the opposite is true when the temperature is increased to 50°C. This temperature dependence is consistent with the hypothesis of stress-induced molecular ordering of the lipids, similar to a phase transition; the cooling limits thermal excitation of the lipid molecules and favor gel-like molecular order in the membrane.

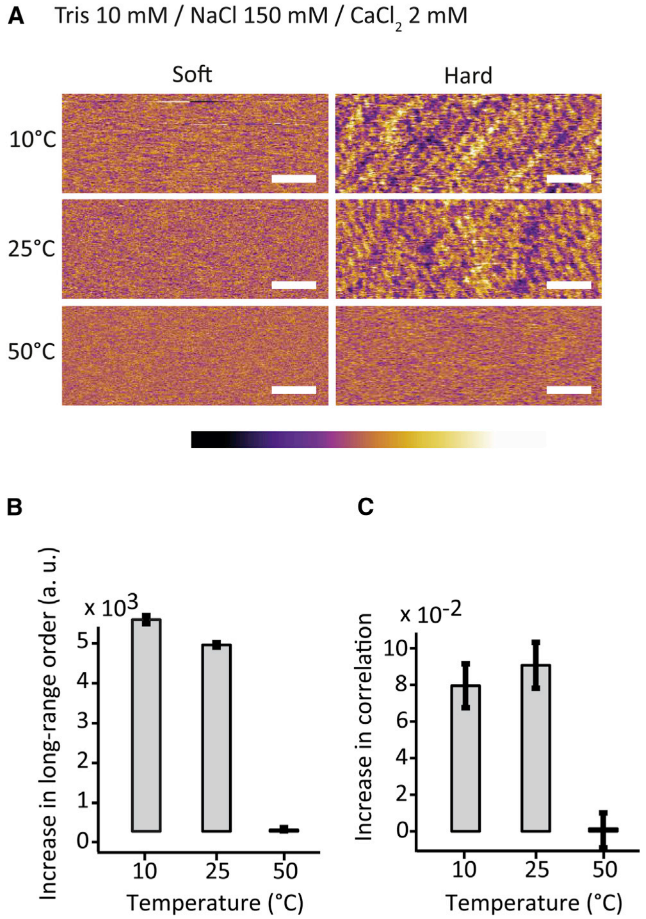

FIGURE 2 AFM images of the evolution of DOPC SLBs on mica as a function of temperature (*A*). The SLB is formed in solution 7 (Tris 10 mM/NaCl 150 mM/$CaCl_2$ 2 mM). The graphs (*B* and *C*) quantify the relative evolution of the stress-induced nanotexture as a function of temperature. The existence of long-range order is given by FFT analysis (*B*) whereas line-by-line correlation (*C*) quantifies short-range order on the membrane (see Materials and Methods and Fig. S4 for details). In all cases, a significant decrease of order is visible at 50°C. Scale bars, 50 nm; color scale bars, height variation of 0.8 nm. To see this figure in color, go online.

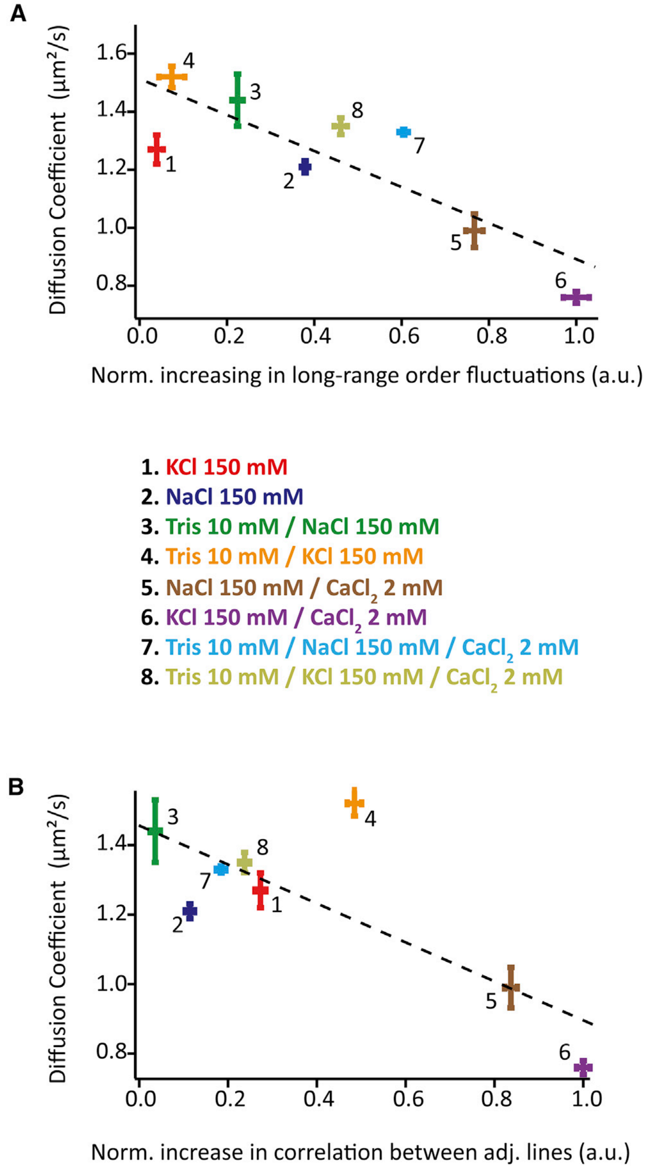

FIGURE 3 Graphs of the lipid diffusion coefficient values in all eight different solutions plotted against the tendency of the membrane to form a nanotexture when under mechanical stress. The two graphs are for the two quantification methods of the stress-induced nanotexturing tendency. The first graph (*A*) gives the diffusion coefficient against the relative increase in long-range (>150 nm) order as the imaging conditions change from Soft to Hard, derived from Fourier analysis. The second graph (*B*) shows the diffusion coefficient against the relative change in spatial correlation between two consecutively acquired line scan profiles as the imaging conditions change from Soft to Hard. The error bars represent the standard error of each measurement. The dotted lines are linear fits of the data. Details of the analysis are given in Materials and Methods and in Figs. S4 and S5.

The tip locally increases the lipid-lipid proximity by momentarily confining the membrane and hence induces local the ordering (similar to gelation) more efficiently. The thermal vibrations of the lipid molecules increase with the temperature, making it more difficult for the confining tip to induce the transition at 50°C.

To analyze more quantitatively the tendency of a given system to undergo local gelation-like effects under stress, we used two different and complementary approaches (see Materials and Methods and Fig. S4 for details). The first approach (Fig. 3 *A*) quantifies the relative increase in long-range order as the imaging conditions change from Soft to Hard. Stable features should allow for improved longer-range order compared to a fluctuating membrane. The quantification is done by integrating the long-range (>150 nm) Fourier intensities in all the directions of the reciprocal space (Fig. S4). The second approach quantifies short-range order by examining the relative average increase in spatial correlation between two consecutive scan profiles upon a change in scanning conditions. If the bilayer is in fluid state, two consecutive lines scan profiles will show

little correlation because surface features are mostly random. However, when gel-like texture appears, the degree of correlation between adjacent profiles significantly increases. Both quantification approaches clearly support the qualitative observations derived from the AFM images.

Nanotexturing versus lipid mobility

Stress and temperature both influence the phase transition in the SLB. The impact of the third parameter central to this study—the ionic content of the solution—is, however, less clear, beyond qualitative observations. It is obvious that ions can enhance or limit nanotexturing, but results can be confusing. For example, temperature studies such as those presented in Fig. 2 did not show conclusive results in certain solutions (Fig. S10). Similar inconclusive observations were made when replacing the phosphocholine zwitterionic lipid headgroup with a negatively charged phosphoserine that strongly binds cations (88,89) (Fig. S11). This apparent complexity suggests that the ions cannot be considered in isolation, but their hydration properties as well as those of the lipids need to be taken into consideration. In any case, it is clear that the mechanisms allowing specific ions or combination of ions to modulate substrate-lipid and lipid-lipid interactions are likely to take place at all times in the solution, and not just when stress is applied with the AFM tip. If a given solution tends to enhance the formation of stress-induced nanotexture, its ions can be expected to stabilize lipid-lipid interactions within the SLB, effectively reducing the mobility of individual lipids molecules and bringing the bilayer closer to a gel transition. As for measurements at different temperatures, the action of the tip only provides a further reduction of mobility that would allow for a local transition similar to gelation to take place. To verify this hypothesis, we conducted FRAP measurements over DOPC SLBs in each of the eight solutions (see Fig. S5 for measurement details). The resulting diffusion coefficients in solution can then be examined against the tendency for stress-induced nanotexture over the same system, quantified using the two approaches described in the previous section (see Materials and Methods and Fig. S4 for details). The result is shown in Fig. 3, where the diffusion coefficient is plotted against the SLB's tendency to form a stress-induced nanotexture, quantified with both analysis methods and in each solution.

Overall, the results shown in Fig. 3 support the hypothesis, namely the existence of an anticorrelation between lipid mobility and the formation of stress-induced nanotexture in the membrane. The low diffusion coefficients obtained in solutions 5 and 6 correlate with the significant texture intensity observed in AFM images under Hard conditions. In contrast, the ternary mixtures that induce the weakest AFM contrast exhibit relative high diffusion coefficients. Monovalent salts such as NaCl and KCl present significant differences in their diffusion coefficients when in association with $CaCl_2$ or with Tris. In any case, these results show that ions are able to locally control the mobility of lipids in a supported membrane also in the absence of mechanical stress or confinement. These observations are not limited to DOPC, and experiments conducted on biologically more relevant POPC bilayers revealed a very similar behavior (see Fig. S12). This confirms the generality of our findings, at least for phosphocholine-based SLBs. The combination of these findings with, previously described, stress-induced nanotexturing effect unveils the possibility of for ion-induced clusters of lipids molecules to exist naturally in the membrane, and act as diffusion units at the mesoscale. In this picture, the AFM only enhances the already existing nanostructure by applying mechanical stress. However, it is not possible to distinguish whether the AFM tip induces or simply reveals the nanotexture solely based on this data. AFM measurements are fundamentally perturbative in nature, and we cannot exclude possible unexpected effects of the AFM tip on the system, especially in the absence of independent experimental confirmation.

Impact of nanotexturing on interacting biomolecules

The results of the last section clearly show that, depending on the ionic composition of the solution between the membrane and its support, the local viscoelastic properties of the bilayer can change significantly. The AFM experiments suggest that this change is not uniform at the molecular level, but rather involves mesoscale lipid clusters on the 20–50 nm range. Practically, the existence of ion-modulated mesoscale variations in fluid bilayers' properties could play an important role in controlling molecular processes in the membrane, for example, spatially guiding dissolved chemicals and biomolecules to certain locations of the membrane, or preventing/enhancing adsorption near supported location. This idea is difficult to verify for natural biomembranes given their complexity, but we tested the underlying principle by examining the spatial details of how cell-penetrating peptides insert into DOPC SLBs exposed to solutions promoting or limiting nanotexturing (solutions 6 and 7, respectively). We use a well-known antimicrobial peptide, Temporin L for this experiment. Temporin L was first isolated from the skin secretion of the European red frog *Rana temporaria* (90,91). Temporin L disrupts plasma membranes inducing cell death in bacteria (92) and parasites (93,94). The results, shown in Fig. 4 confirm the idea: the adsorption of Temporin L significantly differs in solution 6 compared to solution 7, even when imaged in Soft conditions. (Fig. 4, *B* and *D*, respectively). In solution 6, the high degree of similarity between the stress-induced (Fig. 1) and Temporin L-induced (Fig. 4 *B*) nanotextures in the bilayer strongly suggests a correlation: the ionic solution can locally modify the properties of the bilayer,

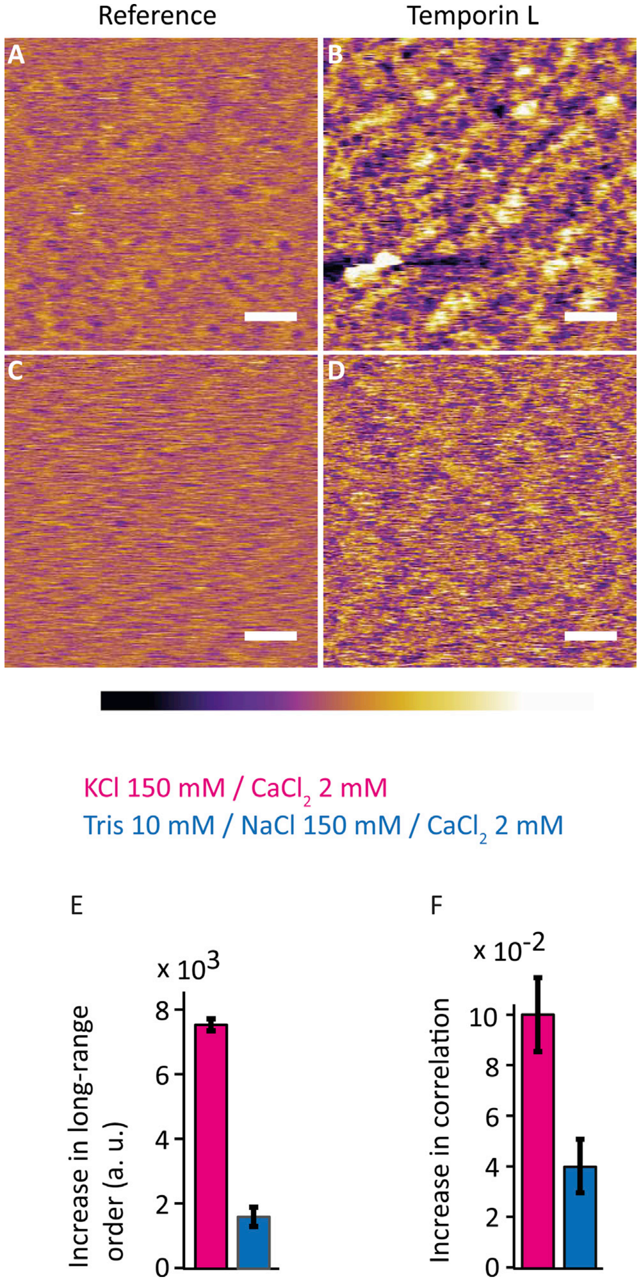

FIGURE 4 Impact of the experimental solution on the adsorption of Temporin L into DOPC SLBs (*A–F*). Two solutions are compared: solution 6 (KCl 150 mM/$CaCl_2$ 2 mM) (*A* and *B*), and solution 7 (Tris 10 mM/NaCl 150 mM/$CaCl_2$ 2 mM) (*C* and *D*). All the AFM images are taken in the Soft imaging condition. The apparition of stable textures in topography is caused by the insertion of Temporin L into the SLB. The process appears dependent on the type of solution in which the experiment takes place. The more pronounced surface texturing is obtained in the solution inducing the largest stress-induced texturing of the pure SLB. In both cases, pH effects can be ruled out (see Materials and Methods). Scale bars, 50 nm; color scale bars, height variation of 0.8 nm. To see this figure in color, go online.

which in turn modulate localize adsorption of the peptide. In contrast, the bilayer structure induced by Temporin L adsorption is less clear in solution 7, despite an increase in long-range order indicating that the membrane is more solidlike (Fig. 4 *D*).

DISCUSSION

Taken together, the results presented in this article suggest that ions can locally order fluid membranes on the mesoscale through interactions involving primarily the lipid headgroups. The effect is particularly strong for SLBs, where ions and water molecules located between the membrane and the support can form a more ordered hydrogen bond network due to spatial confinement (33). The fact that these lipid-ion interactions involve specific ions and are not enhanced with charged phosphoserine headgroups suggest that electrostatics alone is not sufficient to explain the observations, but rather the interacting hydration shells of both the dissolved ions and the headgroups (95). This is further supported by the fact that similar results were obtained with DOPC and POPC SLBs, confirming that interactions between the lipid tails do not dominate the local molecular ordering in the bilayer investigated here. These findings are compatible with previous studies over similar systems where ion-modulated membrane cohesion was found for symmetrically exposed bilayers (85–87,95). Here the results are dominated by the effect of ions on the proximal leaflet, but the tendency nonetheless stands (96,97). The asymmetry induced by substrate interactions can influence the phase-transition temperature of membranes (97), as well as natural membrane fluctuations (96), and lipids microdomain diffusion (98). Our investigations integrate these studies and highlight the specific effect of ion-mediated substrate interactions on the lipids' diffusion and stress-induced nanotexturing. In the absence of mechanical stress, the membrane remains fluid, but with a diffusion coefficient that depends on the ions in solution. We propose that this can be understood as due to the existence of transient lipid clusters, effectively acting as the membrane diffusion units. In this picture, the clusters are held together by the ion-mediated hydration interactions between lipid headgroups with other lipids and the substrate, and their size is on the same order as the spatial texture seen by AFM. Imposing a local stress with the AFM tip does not induce mesoscale molecular ordering; it only reinforces it to the point where local transition similar to gelation can occur over the area scanned by the tip. Practically, this gives rise to the characteristic solidlike nanotexture reported in this article.

Although more work is needed to fully confirm the proposed picture, our results already suggest intriguing possibilities for the function of natural biological membranes. First, the existence of mesoscale order has consequences for the local dynamics induced by the support of fluid membranes; local lipid clusters naturally alter both the mechanical properties of the membrane and its ability to respond to external constraints such as changes in shape. The same mechanisms

could also play a role for the formation of transient proteins and lipid clusters in the membrane, in particular when considering the debated topic of rafts (11,63,84). An important aspect of this picture is its independence on energy, which would make it an efficient amplification mechanism for membrane proteins able to locally induce mechanical stress in the membrane. Second, depending on the ions in contact with the membrane and the supporting structure, chemicals and biomolecules in solution will not interact with each location of the membrane equally, but some spatial modulation will take place. This is exemplified here by the spatially modulated adsorption of a peptide into the DOPC SLB (Fig. 4). Further work will establish the extent to which these effects impact complex natural membranes.

CONCLUSIONS

In this article, we report how different ionic solutions can modulate the nanoscale properties of supported fluid lipid bilayers, allowing local ordering and clustering of the lipid molecules, and promoting stress-induced local gel-like molecular ordering. When observed at the nanoscale, the stress-induced local transitions appear as a nanotexture induced by nodules with a characteristic length scale of 20–30 nm. Combining atomic force microscopy in liquid and FRAP measurements, we correlate the well-known macroscopic effect of ions on lipid mobility in DOPC bilayers with the lipids' tendency to form nanotexture, which we attribute to local ordering in the membrane mediated by the water and ions trapped between the bilayer and its support. Our results highlight a clear but complex interplay among ions, hydration water, substrate, and lipid dynamics at the nanoscale with potentially important consequences for biological membranes. We illustrate this last point by showing that the interaction of peptides with DOPC bilayers is spatially modulated following a similar pattern to the stress-induced nanotexture of the membrane in the same solution in the absence of the peptide. Significantly, we show that ions alone are able to modulate the properties of bilayers over characteristic mesoscopic length scales, resulting in complex membrane behavior without any need for additional energy-dependent processes such as shepherding proteins. We believe our findings could have significant implications for understanding the behavior of biological membranes, in particular the active role played by lipids in supporting biological function.

SUPPORTING MATERIAL

Supporting Materials and Methods and twelve figures are available at http://www.biophysj.org/biophysj/supplemental/S0006-3495(17)30618-5.

AUTHOR CONTRIBUTIONS

L.P. and K.V. conceived the study and designed the experiments. L.P. conducted the measurements. K.V. analyzed the data. H.L.B. and N.R. synthesized the peptide. L.P. and K.V. wrote the manuscript with contributions from S.L.C. and H.L.B.

ACKNOWLEDGMENTS

We thank Dr. Amir Farokh Payam and William Trewby (Physics Department, University of Durham) for help with mathematical analysis and Ethan J. Miller (Physics Department, University of Durham) for his assistance in the FRAP experiments.

This research was supported by the Engineering and Physical Sciences Research Council (grant EP/M023915/1), the Biotechnology and Biological Sciences Research Council (grant BB/M024830/1), and Durham University EPSRC DTG (H.L.B.).

SUPPORTING CITATIONS

References (99–104) appear in the Supporting Material.

REFERENCES

1. Cooper, G. M., and R. E. Hausman. 2016. The Cell: A Molecular Approach, 7th Ed. Sinauer Associates, Sunderland, MA.
2. Singer, S. J., and G. L. Nicolson. 1972. The fluid mosaic model of the structure of cell membranes. *Science.* 175:720–731.
3. Bagatolli, L. A., J. H. Ipsen, …, O. G. Mouritsen. 2010. An outlook on organization of lipids in membranes: searching for a realistic connection with the organization of biological membranes. *Prog. Lipid Res.* 49:378–389.
4. Carquin, M., L. D'Auria, …, D. Tyteca. 2016. Recent progress on lipid lateral heterogeneity in plasma membranes: from rafts to submicrometric domains. *Prog. Lipid Res.* 62:1–24.
5. Chiantia, S., and E. London. 2012. Acyl chain length and saturation modulate interleaflet coupling in asymmetric bilayers: effects on dynamics and structural order. *Biophys. J.* 103:2311–2319.
6. Nickels, J. D., J. C. Smith, and X. Cheng. 2015. Lateral organization, bilayer asymmetry, and inter-leaflet coupling of biological membranes. *Chem. Phys. Lipids.* 192:87–99.
7. Zimmerberg, J., and M. M. Kozlov. 2006. How proteins produce cellular membrane curvature. *Nat. Rev. Mol. Cell Biol.* 7:9–19.
8. van Meer, G., D. R. Voelker, and G. W. Feigenson. 2008. Membrane lipids: where they are and how they behave. *Nat. Rev. Mol. Cell Biol.* 9:112–124.
9. Gambin, Y., M. Reffay, …, W. Urbach. 2010. Variation of the lateral mobility of transmembrane peptides with hydrophobic mismatch. *J. Phys. Chem. B.* 114:3559–3566.
10. Pincet, F., V. Adrien, …, D. Tareste. 2016. FRAP to characterize molecular diffusion and interaction in various membrane environments. *PLoS One.* 11:e0158457.
11. Jacobson, K., O. G. Mouritsen, and R. G. W. Anderson. 2007. Lipid rafts: at a crossroad between cell biology and physics. *Nat. Cell Biol.* 9:7–14.
12. Cremer, P. S., and S. G. Boxer. 1999. Formation and spreading of lipid bilayers on planar glass supports. *J. Phys. Chem. B.* 103:2554–2559.
13. Richter, R., A. Mukhopadhyay, and A. Brisson. 2003. Pathways of lipid vesicle deposition on solid surfaces: a combined QCM-D and AFM study. *Biophys. J.* 85:3035–3047.
14. Akesson, A., T. Lind, …, M. Cardenas. 2012. Composition and structure of mixed phospholipid supported bilayers formed by POPC and DPPC. *Soft Matter.* 8:5658–5665.
15. Veatch, S. L., and S. L. Keller. 2003. Separation of liquid phases in giant vesicles of ternary mixtures of phospholipids and cholesterol. *Biophys. J.* 85:3074–3083.

16. Ashkar, R., M. Nagao, …, T. Koga. 2015. Tuning membrane thickness fluctuations in model lipid bilayers. *Biophys. J.* 109:106–112.

17. Bradley, R. P., and R. Radhakrishnan. 2016. Curvature-undulation coupling as a basis for curvature sensing and generation in bilayer membranes. *Proc. Natl. Acad. Sci. USA.* 113:E5117–E5124.

18. Johnson, S. J., T. M. Bayerl, …, E. Sackmann. 1991. Structure of an adsorbed dimyristoylphosphatidylcholine bilayer measured with specular reflection of neutrons. *Biophys. J.* 59:289–294.

19. Picas, L., P. E. Milhiet, and J. Hernández-Borrell. 2012. Atomic force microscopy: a versatile tool to probe the physical and chemical properties of supported membranes at the nanoscale. *Chem. Phys. Lipids.* 165:845–860.

20. Machán, R., and M. Hof. 2010. Lipid diffusion in planar membranes investigated by fluorescence correlation spectroscopy. *Biochim. Biophys. Acta.* 1798:1377–1391.

21. Kam, L. C. 2009. Capturing the nanoscale complexity of cellular membranes in supported lipid bilayers. *J. Struct. Biol.* 168:3–10.

22. Melzak, K. A., S. Moreno-Flores, …, J. L. Toca-Herrera. 2011. Why size and speed matter: frequency dependence and the mechanical properties of biomolecules. *Soft Matter.* 7:332–342.

23. Wu, H.-L., H.-K. Tsao, and Y.-J. Sheng. 2016. Dynamic and mechanical properties of supported lipid bilayers. *J. Chem. Phys.* 144:154904.

24. Giménez, D., O. L. Sánchez-Muñoz, and J. Salgado. 2015. Direct observation of nanometer-scale pores of melittin in supported lipid monolayers. *Langmuir.* 31:3146–3158.

25. Rapson, A. C., M. A. Hossain, …, M. L. Gee. 2011. Structural dynamics of a lytic peptide interacting with a supported lipid bilayer. *Biophys. J.* 100:1353–1361.

26. Wu, J.-C., P.-Y. Tseng, …, Y.-C. Chang. 2013. Antibody conjugated supported lipid bilayer for capturing and purification of viable tumor cells in blood for subsequent cell culture. *Biomaterials.* 34:5191–5199.

27. Lee, Y. K., S. Kim, and J.-M. Nam. 2015. Dark-field-based observation of single-nanoparticle dynamics on a supported lipid bilayer for in situ analysis of interacting molecules and nanoparticles. *ChemPhysChem.* 16:77–84.

28. Horvath, R., B. Kobzi, …, E. Kiss. 2013. Molecular interaction of a new antibacterial polymer with a supported lipid bilayer measured by an in situ label-free optical technique. *Int. J. Mol. Sci.* 14:9722–9736.

29. Sarangi, N. K., and A. Patnaik. 2012. L-tryptophan-induced electron transport across supported lipid bilayers: an alkyl-chain tilt-angle, and bilayer-symmetry dependence. *ChemPhysChem.* 13:4258–4270.

30. Becucci, L., D. Valensin, …, R. Guidelli. 2014. Dermcidin, an anionic antimicrobial peptide: influence of lipid charge, pH and Zn^{2+} on its interaction with a biomimetic membrane. *Soft Matter.* 10:616–626.

31. Jacobson, K., and D. Papahadjopoulos. 1975. Phase transitions and phase separations in phospholipid membranes induced by changes in temperature, pH, and concentration of bivalent cations. *Biochemistry.* 14:152–161.

32. Anderson, T. H., Y. Min, …, J. N. Israelachvili. 2009. Formation of supported bilayers on silica substrates. *Langmuir.* 25:6997–7005.

33. Tero, R. 2012. Substrate effects on the formation process, structure and physicochemical properties of supported lipid bilayers. *Materials.* 5:2658–2680.

34. Dekkiche, F., M. C. Corneci, …, J. P. Rieu. 2010. Stability and tribological performances of fluid phospholipid bilayers: effect of buffer and ions. *Colloids Surf. B Biointerfaces.* 80:232–239.

35. Garcia-Manyes, S., G. Oncins, and F. Sanz. 2006. Effect of pH and ionic strength on phospholipid nanomechanics and on deposition process onto hydrophilic surfaces measured by AFM. *Electrochim. Acta.* 51:5029–5036.

36. Böckmann, R. A., A. Hac, …, H. Grubmüller. 2003. Effect of sodium chloride on a lipid bilayer. *Biophys. J.* 85:1647–1655.

37. Mao, Y., Y. Du, …, H. Jiang. 2013. Binding competition to the POPG lipid bilayer of Ca^{2+}, Mg^{2+}, Na^{+}, and K^{+} in different ion mixtures and biological implication. *J. Phys. Chem. B.* 117:850–858.

38. Gurtovenko, A. A., and I. Vattulainen. 2008. Effect of NaCl and KCl on phosphatidylcholine and phosphatidylethanolamine lipid membranes: insight from atomic-scale simulations for understanding salt-induced effects in the plasma membrane. *J. Phys. Chem. B.* 112:1953–1962.

39. Lind, T. K., M. Cárdenas, and H. P. Wacklin. 2014. Formation of supported lipid bilayers by vesicle fusion: effect of deposition temperature. *Langmuir.* 30:7259–7263.

40. Reimhult, E., F. Hook, and B. Kasemo. 2003. Intact vesicle adsorption and supported biomembrane formation from vesicles in solution: influence of surface chemistry, vesicle size, temperature, and osmotic pressure. *Langmuir.* 19:1681–1691.

41. Head, B. P., H. H. Patel, and P. A. Insel. 2014. Interaction of membrane/lipid rafts with the cytoskeleton: impact on signaling and function: membrane/lipid rafts, mediators of cytoskeletal arrangement and cell signaling. *Biochim. Biophys. Acta.* 1838:532–545.

42. Zhong, J., and J. Yan. 2016. Seeing is believing: atomic force microscopy imaging for nanomaterial research. *RSC Adv.* 6:1103–1121.

43. Takeshi, F. 2009. Subnanometer-resolution frequency modulation atomic force microscopy in liquid for biological applications. *Jpn. J. Appl. Phys.* 48:08JA01.

44. Miller, E. J., W. Trewby, …, K. Voïtchovsky. 2016. Sub-nanometer resolution imaging with amplitude-modulation atomic force microscopy in liquid. *J. Vis. Exp.* 118. http://dx.doi.org/10.3791/54924.

45. Giocondi, M. C., D. Yamamoto, …, C. Le Grimellec. 2010. Surface topography of membrane domains. *Biochim. Biophys. Acta.* 1798:703–718.

46. Hohlbauch, S. 2016. Nanomechanical and viscoelastic measurements in biological atomic force microscopy (AFM). *Biophys. J.* 110:498a–499a.

47. Dufrene, Y. F., and G. U. Lee. 2000. Advances in the characterization of supported lipid films with the atomic force microscope. *Biochim. Biophys. Acta.* 1509:14–41.

48. Chiantia, S., N. Kahya, …, P. Schwille. 2006. Effects of ceramide on liquid-ordered domains investigated by simultaneous AFM and FCS. *Biophys. J.* 90:4500–4508.

49. Chiang, Y.-L., Y.-C. Chang, …, Y.-L. Shih. 2015. Atomic force microscopy characterization of protein fibrils formed by the amyloidogenic region of the bacterial protein minE on mica and a supported lipid bilayer. *PLoS One.* 10:e0142506.

50. Ricci, M., R. A. Quinlan, and K. Voïtchovsky. 2016. Sub-nanometre mapping of the aquaporin-water interface using multifrequency atomic force microscopy. *Soft Matter.* 13:187–195.

51. Fukuma, T., M. J. Higgins, and S. P. Jarvis. 2007. Direct imaging of individual intrinsic hydration layers on lipid bilayers at Ångstrom resolution. *Biophys. J.* 92:3603–3609.

52. Antoranz Contera, S., K. Voïtchovsky, and J. F. Ryan. 2010. Controlled ionic condensation at the surface of a native extremophile membrane. *Nanoscale.* 2:222–229.

53. Fukuma, T., M. J. Higgins, and S. P. Jarvis. 2007. Direct imaging of lipid-ion network formation under physiological conditions by frequency modulation atomic force microscopy. *Phys. Rev. Lett.* 98:106101.

54. Ricci, M., P. Spijker, …, K. Voïtchovsky. 2013. Direct visualization of single ions in the Stern layer of calcite. *Langmuir.* 29:2207–2216.

55. Ricci, M., W. Trewby, …, K. Voïtchovsky. 2017. Direct observation of the dynamics of single metal ions at the interface with solids in aqueous solutions. *Sci. Rep.* 7:43234.

56. Voïtchovsky, K., J. J. Kuna, …, F. Stellacci. 2010. Direct mapping of the solid-liquid adhesion energy with subnanometre resolution. *Nat. Nanotechnol.* 5:401–405.

57. Evert, L. L., D. Leckband, and J. N. Israelachvili. 1994. Structure and dynamics of ion-induced domains in free and supported monolayers and bilayers. *Langmuir.* 10:303–315.

58. Garcia-Manyes, S., G. Oncins, and F. Sanz. 2005. Effect of ion-binding and chemical phospholipid structure on the nanomechanics of lipid bilayers studied by force spectroscopy. *Biophys. J.* 89:1812–1826.

59. Dickey, A., and R. Faller. 2008. Examining the contributions of lipid shape and headgroup charge on bilayer behavior. *Biophys. J.* 95:2636–2646.

60. Marra, J., and J. Israelachvili. 1985. Direct measurements of forces between phosphatidylcholine and phosphatidylethanolamine bilayers in aqueous electrolyte solutions. *Biochemistry.* 24:4608–4618.

61. Bouvrais, H., L. Duelund, and J. H. Ipsen. 2014. Buffers affect the bending rigidity of model lipid membranes. *Langmuir.* 30:13–16.

62. Cevc, G., A. Watts, and D. Marsh. 1981. Titration of the phase transition of phosphatidylserine bilayer membranes. Effects of pH, surface electrostatics, ion binding, and head-group hydration. *Biochemistry.* 20:4955–4965.

63. Dietrich, C., L. A. Bagatolli, …, E. Gratton. 2001. Lipid rafts reconstituted in model membranes. *Biophys. J.* 80:1417–1428.

64. Mingeot-Leclercq, M.-P., M. Deleu, …, Y. F. Dufrêne. 2008. Atomic force microscopy of supported lipid bilayers. *Nat. Protoc.* 3:1654–1659.

65. Ricci, M., P. Spijker, and K. Voïtchovsky. 2014. Water-induced correlation between single ions imaged at the solid-liquid interface. *Nat. Commun.* 5:4400.

66. Butt, H. J., and M. Jaschke. 1995. Calculation of thermal noise in atomic force microscopy. *Nanotechnology.* 6:1.

67. Horcas, I., R. Fernández, …, A. M. Baro. 2007. WSXM: a software for scanning probe microscopy and a tool for nanotechnology. *Rev. Sci. Instrum.* 78:013705.

68. Kang, M., C. A. Day, …, E. DiBenedetto. 2012. Simplified equation to extract diffusion coefficients from confocal FRAP data. *Traffic.* 13:1589–1600.

69. Blumenthal, D., L. Goldstien, …, L. A. Gheber. 2015. Universal approach to FRAP analysis of arbitrary bleaching patterns. *Sci. Rep.* 5:11655.

70. Kunze, A., F. Zhao, …, B. Kasemo. 2011. Ion-mediated changes of supported lipid bilayers and their coupling to the substrate. A case of bilayer slip? *Soft Matter.* 7:8582–8591.

71. Voïtchovsky, K. 2013. Anharmonicity, solvation forces, and resolution in atomic force microscopy at the solid-liquid interface. *Phys. Rev. E Stat. Nonlin. Soft Matter Phys.* 88:022407.

72. Anczykowski, B., D. Krüger, …, H. Fuchs. 1996. Basic properties of dynamic force spectroscopy with the scanning force microscope in experiment and simulation. *Ultramicroscopy.* 66:251–259.

73. Adamcik, J., A. Berquand, and R. Mezzenga. 2011. Single-step direct measurement of amyloid fibrils stiffness by peak force quantitative nanomechanical atomic force microscopy. *Appl. Phys. Lett.* 98:193701.

74. Rico, F., C. Su, and S. Scheuring. 2011. Mechanical mapping of single membrane proteins at submolecular resolution. *Nano Lett.* 11:3983–3986.

75. Voïtchovsky, K., S. Antoranz Contera, …, J. F. Ryan. 2006. Differential stiffness and lipid mobility in the leaflets of purple membranes. *Biophys. J.* 90:2075–2085.

76. Perkins, M., S. J. Ebbens, …, N. Patel. 2007. Elastic modulus measurements from individual lactose particles using atomic force microscopy. *Int. J. Pharm.* 332:168–175.

77. Phillips, R., T. Ursell, …, P. Sens. 2009. Emerging roles for lipids in shaping membrane-protein function. *Nature.* 459:379–385.

78. Seantier, B., and B. Kasemo. 2009. Influence of mono- and divalent ions on the formation of supported phospholipid bilayers via vesicle adsorption. *Langmuir.* 25:5767–5772.

79. Trewby, W., D. Livesey, and K. Voïtchovsky. 2016. Buffering agents modify the hydration landscape at charged interfaces. *Soft Matter.* 12:2642–2651.

80. Alessandrini, A., and P. Facci. 2014. Phase transitions in supported lipid bilayers studied by AFM. *Soft Matter.* 10:7145–7164.

81. Li, M., M. Chen, …, J. Lipkowski. 2008. AFM studies of solid-supported lipid bilayers formed at an Au(111) electrode surface using vesicle fusion and a combination of Langmuir-Blodgett and Langmuir-Schaefer techniques. *Langmuir.* 24:10313–10323.

82. Janiak, M. J., D. M. Small, and G. G. Shipley. 1976. Nature of the Thermal pretransition of synthetic phospholipids: dimyristoyl- and dipalmitoyl lecithin. *Biochemistry.* 15:4575–4580.

83. Cevc, G. 1991. Polymorphism of the bilayer membranes in the ordered phase and the molecular origin of the lipid pretransition and rippled lamellae. *Biochim. Biophys. Acta.* 1062:59–69.

84. Leonenko, Z. V., E. Finot, …, D. T. Cramb. 2004. Investigation of temperature-induced phase transitions in DOPC and DPPC phospholipid bilayers using temperature-controlled scanning force microscopy. *Biophys. J.* 86:3783–3793.

85. Redondo-Morata, L., M. I. Giannotti, and F. Sanz. 2014. Structural impact of cations on lipid bilayer models: nanomechanical properties by AFM-force spectroscopy. *Mol. Membr. Biol.* 31:17–28.

86. Redondo-Morata, L., G. Oncins, and F. Sanz. 2012. Force spectroscopy reveals the effect of different ions in the nanomechanical behavior of phospholipid model membranes: the case of potassium cation. *Biophys. J.* 102:66–74.

87. Garcia-Manyes, S., L. Redondo-Morata, …, F. Sanz. 2010. Nanomechanics of lipid bilayers: heads or tails? *J. Am. Chem. Soc.* 132:12874–12886.

88. Martín-Molina, A., C. Rodríguez-Beas, and J. Faraudo. 2012. Effect of calcium and magnesium on phosphatidylserine membranes: experiments and all-atomic simulations. *Biophys. J.* 102:2095–2103.

89. Rodríguez, Y., M. Mezei, and R. Osman. 2007. Association free energy of dipalmitoyl phosphatidylserines in a mixed dipalmitoylphosphatidylcholine membrane. *Biophys. J.* 92:3071–3080.

90. Rinaldi, A. C., M. L. Mangoni, …, M. Simmaco. 2002. Temporin L: antimicrobial, haemolytic and cytotoxic activities, and effects on membrane permeabilization in lipid vesicles. *Biochem. J.* 368:91–100.

91. Simmaco, M., G. Mignogna, …, D. Barra. 1996. Temporins, antimicrobial peptides from the European red frog Rana temporaria. *Eur. J. Biochem.* 242:788–792.

92. Mangoni, M. L., N. Papo, …, A. C. Rinaldi. 2004. Effects of the antimicrobial peptide temporin L on cell morphology, membrane permeability and viability of *Escherichia coli*. *Biochem. J.* 380:859–865.

93. Cobb, S. L., and P. W. Denny. 2010. Antimicrobial peptides for leishmaniasis. *Curr. Opin. Invest. Drugs.* 11:868–875.

94. Eggimann, G. A., K. Sweeney, …, P. W. Denny. 2015. The role of phosphoglycans in the susceptibility of Leishmania mexicana to the temporin family of anti-microbial peptides. *Molecules.* 20:2775–2785.

95. Melcrová, A., S. Pokorna, …, L. Cwiklik. 2016. The complex nature of calcium cation interactions with phospholipid bilayers. *Sci. Rep.* 6:38035.

96. Duro, N., M. Gjika, …, S. Varma. 2016. POPC bilayers supported on nanoporous substrates: specific effects of silica-type surface hydroxylation and charge density. *Langmuir.* 32:6766–6774.

97. Seeger, H. M., G. Marino, …, P. Facci. 2009. Effect of physical parameters on the main phase transition of supported lipid bilayers. *Biophys. J.* 97:1067–1076.

98. Sterling, S. M., R. Dawes, …, D. J. Neivandt. 2015. Comparison of actin- and glass-supported phospholipid bilayer diffusion coefficients. *Biophys. J.* 108:1946–1953.

99. Müller, D. J., M. Amrein, and A. Engel. 1997. Adsorption of biological molecules to a solid support for scanning probe microscopy. *J. Struct. Biol.* 119:172–188.

100. Veatch, S. L., and S. L. Keller. 2002. Organization in lipid membranes containing cholesterol. *Phys. Rev. Lett.* 89:268101.

101. Stevens, M. M., A. R. Honerkamp-Smith, and S. L. Keller. 2010. Solubility limits of cholesterol, lanosterol, ergosterol, stigmasterol, and β-sitosterol in electroformed lipid vesicles. *Soft Matter.* 6:5882–5890.

102. Ma, Y., S. K. Ghosh, …, S. K. Sinha. 2016. Cholesterol partition and condensing effect in phase-separated ternary mixture lipid multilayers. *Biophys. J.* 110:1355–1366.

103. Almeida, P. F. 2011. A simple thermodynamic model of the liquid-ordered state and the interactions between phospholipids and cholesterol. *Biophys. J.* 100:420–429.

104. Gumi-Audenis, B., L. Costa, …, M. I. Giannotti. 2016. Structure and nanomechanics of model membranes by atomic force microscopy and spectroscopy: insights into the role of cholesterol and sphingolipids. *Membranes (Basel).* 6:58.

Biophysical Journal, Volume *113*

Supplemental Information

Ions Modulate Stress-Induced Nanotexture in Supported Fluid Lipid Bilayers

Luca Piantanida, Hannah L. Bolt, Neshat Rozatian, Steven L. Cobb, and Kislon Voïtchovsky

Supporting Material

Ions modulate stress-induced nano-texture in supported fluid lipid bilayers

Luca Piantanida[1], Hannah L. Bolt[2], Neshat Rozatian[2], Steven L. Cobb[2], and Kislon Voïtchovsky[1*]

[1]Department of Physics and [2]Department of Chemistry, Durham University, South Road, Durham, DH1 3LE, UK.

TABLE OF CONTENT OF THE SUPPORTING MATERIAL

1. Impact of salt concentration: Deposition vs Imaging solution
2. Temporin L characterisation data
3. Influence of the tip curvature radius
4. Analysis methods for quantification of the membrane texture
5. Fluorescence recovery after photobleaching (FRAP) analysis
6. Reversibility of the tip-induced nano-texturing
7. Cholesterol structuring effect
8. Comparison of imaging modes: contact vs amplitude modulation
9. Analysis of a DOPC bilayer on silicon oxide
10. Effect of temperature on DOPC with solution 4
11. Effect of temperature on DOPS SLBs
12. Comparison of texture formation on different lipids (DOPC and POPC)
13. Supporting references

1. IMPACT OF SALT CONCENTRATION: DEPOSITION VS IMAGING SOLUTION

The saline concentration of the solution used to create the SLBs (formation solution) is 15 times higher than that of the solution used for imaging. It is well known that ions play an important role for vesicle deposition and fusion when forming SLBs (1, 5). Ions influence the lipid-lipid interactions as well as the hydration state of lipid headgroups and mediate, interactions between the proximal leaflet and the substrate. The study examines the impact of ions on the nanoscale behaviour of SLBs. To emphasise effects related to the supporting structure, we rinsed fully formed membranes with a diluted solution so as to limit ionic effects in the distal leaflet by comparison with the proximal where ions are partially trapped between the membrane and the substrate. Figure S1 shows a comparison between supported lipid bilayer formed in solution 7 (Tris 10 mM / NaCl 150 mM / $CaCl_2$ 2 mM) and imaged in four different dilutions of the deposition solution (undiluted, 5×, 10×, and 15×). Generally, the dilution does not prevent the apparition of nano-texture but emphasises the effect of tip-induced stress. At full concentration (undiluted), some texture is clearly visible in Medium imaging conditions already (see Materials and Methods for a definition of the imaging conditions). As the imaging solution is diluted, the effect becomes less intense and only occurs under Hard conditions, but its aspect remain similar in all imaging conditions. Using diluted solutions not only enhances the effect of ion-mediated interaction with the substrate, but it also enables working with strongly texturing solution (e.g. solution 6, see Fig. 1). We applied this protocol to all the imaging experiment (AFM and FRAP) in order to give a complete comparison of the data.

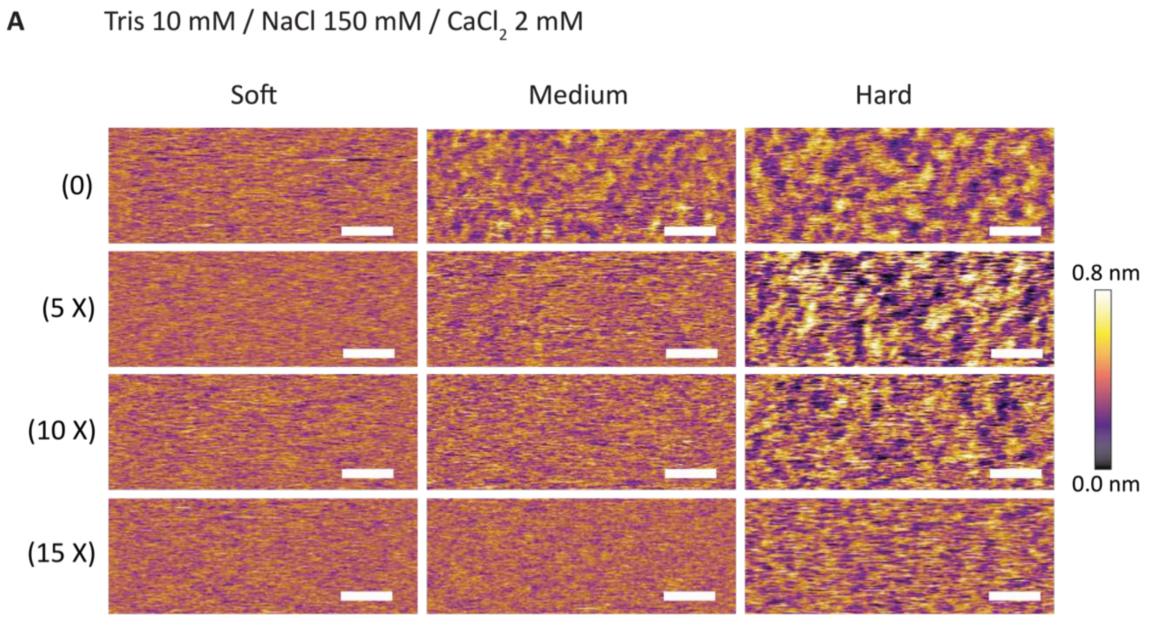

FIGURE S1. Comparison of representative AFM images of SLBs formed in different dilutions of solution 7 (Tris 10 mM / NaCl 150 mM / $CaCl_2$ 2 mM) under Soft, Medium and Hard imaging conditions (see Materials and Methods for details on the imaging parameters). The membrane is always formed in solution 7, but the sample is then rinsed with either undiluted, 5×, 10×, or 15× diluted solution 7 before the AFM measurement. Stress-induced texture is visible all cases, but its intensity decreases with diluting the solution. The scale bar is 50 nm in all images.

2. TEMPORIN L CHARACTERISATION DATA

QToF MS

Mass spectroscopy measurements were performed using a QToF Premier mass spectrometer with an Acquity ultra-performance liquid chromatography system (Waters Ltd, UK). Samples were injected to the Acquity UPLC BEH C18 column (1.7 µm, 2.1 mm x 100 mm) with a flow rate of 0.6 mL min^{-1} and a linear gradient of 0–99 % of solvent B over 6 min (A = 0.1 % formic acid in H_2O, B = 0.1 % formic acid in acetonitrile). The solvent flow from the UPLC was injected into a 0.2 mL/min flow of acetonitrile which was introduced into the electrospray ion source. Temporin L mass calculated for *m*/*z* $[M+H]^+$ 821.0, mass observed $[M+2H]^{2+}$ 820.7

Analytical HPLC

Samples were dissolved in 100 µL acidified water and the purity of products was estimated by an injection of 10 µL to analytical RP-HPLC using a Perkin Elmer 200 Series LC pump with a Perkin-Elmer 785A UV-vis detector on an SB Analytical column (ODS-H Optimal), 4.6 x 100mm , 3.5 µm; flow rate = 1 mL min^{-1}; loop size = 20 µL; column oven 40 ˚C; λ = 220 nm; gradient: 0–100 % solvent B over 30 min (solvent A: 95 % H_2O, 5 %, MeCN, 0.05 % TFA; solvent B: 95 %, MeCN, 5 % H_2O, 0.03 % TFA). Temporin L was found by RP-analytical HPLC RT 17.8 min with approximate purity > 99 %.

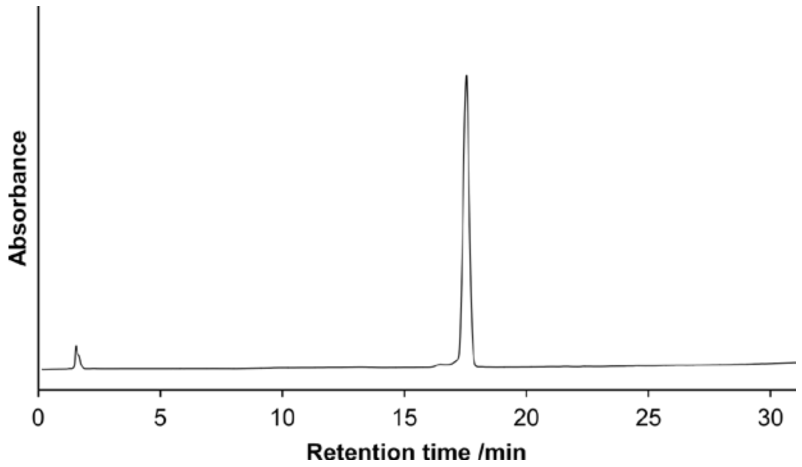

FIGURE S2. Analytical RP-HPLC chromatogram for Temporin L peptide. Flow rate = 1 mL min^{-1}; loop size = 20 µL; column oven 40 ˚C; λ = 220 nm; gradient: 0–100 % solvent B over 30 min (solvent A: 95 % H_2O, 5 %, MeCN, 0.05 % TFA; solvent B: 95 %, MeCN, 5 % H_2O, 0.03 % TFA).

3. INFLUENCE OF THE TIP CURVATURE RADIUS

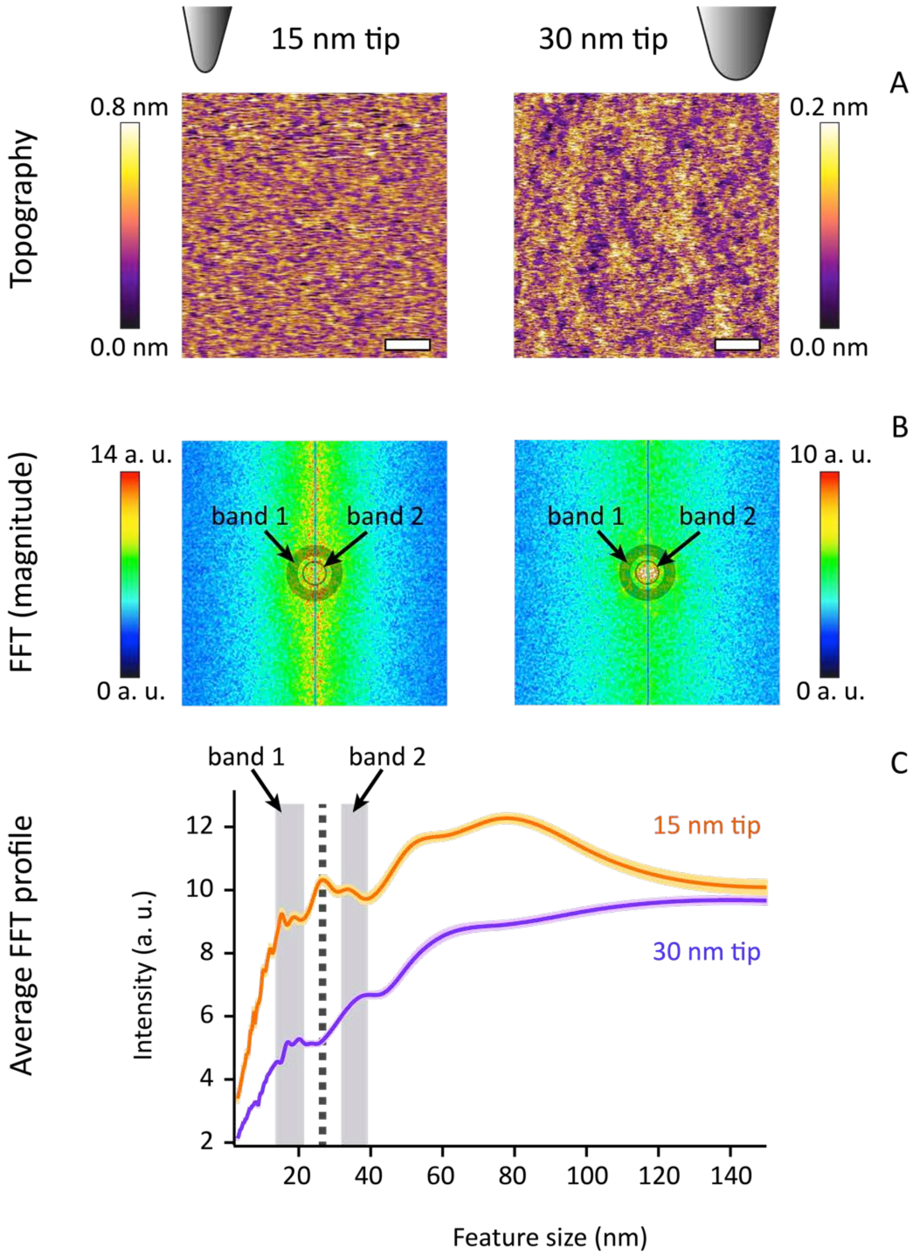

FIGURE S3. Comparison of the stress-induced features obtained with tips exhibiting different radii. The sharper tip has a maximum tip radius of 15 nm (Olympus, RC800PSA). The blunter tip is rounded by design to a 30 nm radius (Nanosensor, SD-R30-FM). Both tips are composed of silicon oxide. Topographic images were acquired in Hard imaging conditions with each tip over a same sample (A). Qualitatively, the images both exhibit some texture, but some differences are visible. First the height variations are more important with the sharper tip, reflecting the higher local pressures exerted by the tip on the membrane. To quantify similarities and differences, Fourier analysis was performed on both images (B-C). Two bands (grey area in B and C) highlight local maxima corresponding to features of size ~20 nm and ~35 nm that are present in the 2D FFT derived with both tips. This suggests that the nano-texture is an intrinsic property of the DOPC membrane on mica, rather than a reflection of the tip geometry. There are, however, some differences highlighted by the black dotted line in (C). Small differences are to be expected if the texture is induced by a local fluid to gel-like transition promoted by the tip pressure, but the existence of ~20 nm nodules suggest an ideal packing arrangement for the lipid molecules under pressure. The scale bar in (A) is 50 nm and the imaging is conducted on a DOPC bilayer immersed in solution 7 (Tris 10 mM / NaCl 150 mM / $CaCl_2$ 2 mM).

4. ANALYSIS METHODS FOR QUANTIFICATION OF THE MEMBRANE TEXTURE

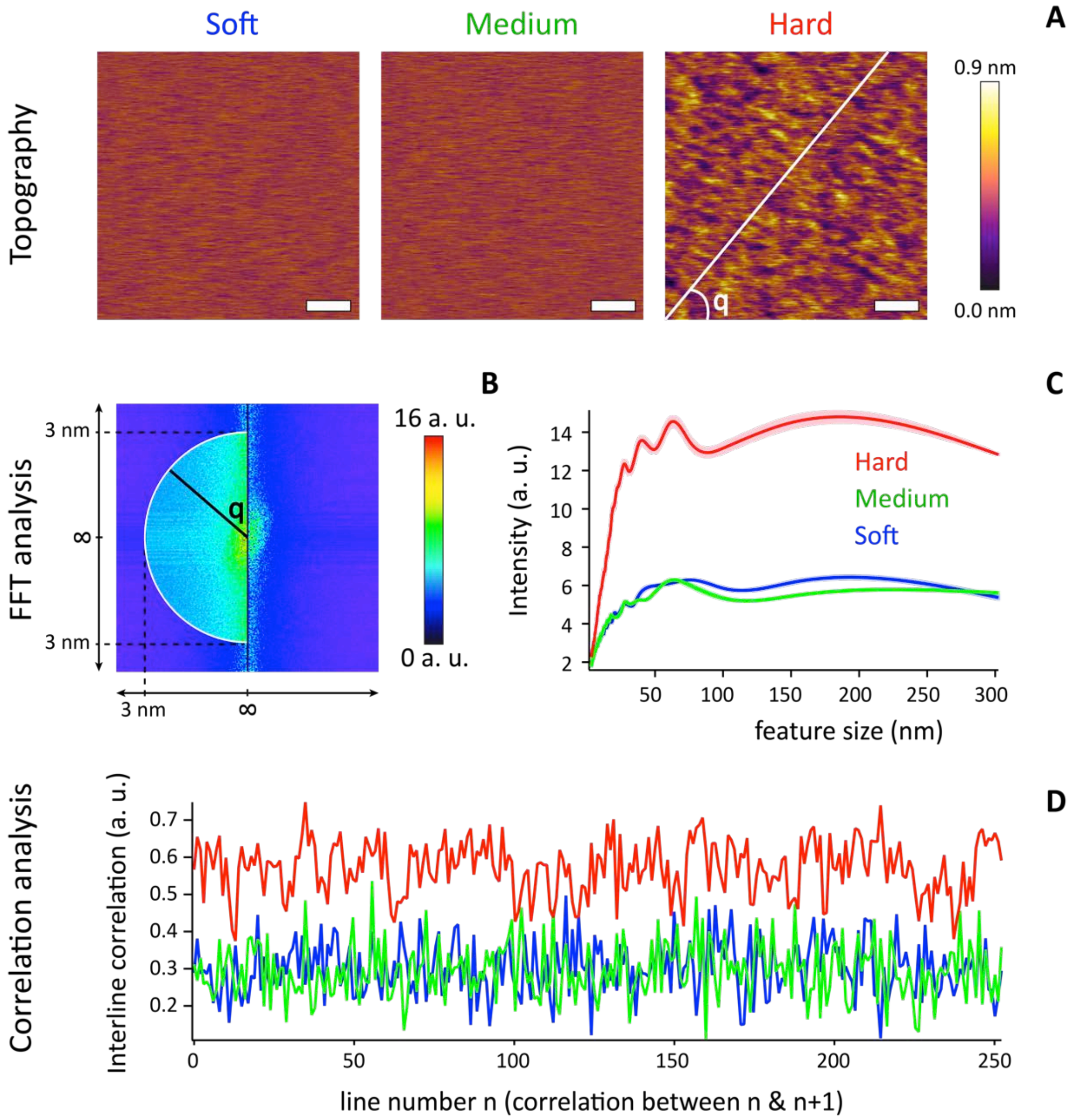

FIGURE S4. Analysis methods used to quantitatively process the stress-induced texturing of bilayers, illustrated here for DOPC immersed in solution 7 (Tris 10 mM / NaCl 150 mM / $CaCl_2$ 2 mM). Qualitatively, the topographic images (A) reveal little change between Soft and Medium imaging conditions, but a dramatic change occurs in Hard conditions. The 2D FFT of the image acquired in Hard conditions is shown in (B). The black line is the 1D FFT of the profile shown in white in (A, Hard). It covers features with sizes between 3 nm and infinity at the centre of the image (reciprocal space). The profile is then averaged over all directions (all θ angles, with 90° < θ < 270°) and the average converted back to real space (C). The converted averaged profiles are given in (C) for each imaging conditions, with the shading representing the standard error. The intensity is significantly higher for the Hard imaging conditions while the Soft and Medium conditions are similar, reflecting the qualitative observations of the height images. The quantitative increase in long-range order is subsequently calculated by integration of the Fourier profiles over a selected interval of feature sizes (see text). The correlation analysis (D) examines the degree of correlation between each set of 2 adjacent scan profiles. Since each image is composed of 256 lines, 254 pairs (correlation values) are obtained with each value between 0 (no correlation at all) and 1 (identical profiles) (curves in D). The degree of correlation is overall higher in for the Hard imaging conditions as expected from the topographic images. A single correlation value is obtained for each image by averaging the 255 correlation values and taking its standard error. The Scale bar in (A) is 50 nm.

5. FLUORESCENCE RECOVERY AFTER PHOTOBLEACHING (FRAP) ANALYSIS

The lipids diffusion coefficient was calculated for DOPC immersed in each of the solutions investigated (Table 1). The measurements were performed using FRAP analysis (see Materials and Methods). Since our bleaching pattern is a square, we adapted the derivation given in reference (2) for the fluorescence intensity $I(t)$ as follow:

$$I(t) = a_0 + a_1\left(1 - \sqrt{\frac{w^2}{w^2 + 4\pi D(t - t_{bleach})}}\right) \qquad (s1)$$

where t is time, the spot area geometry is represented by the square width w (μm) and D ($\mu m^2/s$) is the diffusion coefficient. The acquired sequence coincides with the start of fluorescence recovery where t_{bleach} is zero. Before analysis, the recovery spectra are normalised using two parameters: the offset (a_0) and the amplitude (a_1) of the recorded intensity. The diffusion coefficients used in the paper are an average of at least three different sequences captured, and the associate uncertainty corresponds to one standard deviation. Higher uncertainties are found when the SLB patches exhibit significantly size variations (Fig. S5 F).

Figure S4 shows an extract of a typical FRAP experiments. Four representative frames are displayed (Fig. S5 B-E) with sequential fluorescence recovery of the bleached region (red arrow). The fluorescence intensity of the bleached area is calculated by averaging the mean intensity value of all pixels of the area (red arrow). The fluidity of the lipids in the SLB produces fluctuations in the intensity value that can be seen in the example intensity profiles shown immediately under each frame (Fig. S5 B-E). Un-fused lipid vesicles induce the brighter background 'spots' in the florescence images, but this can be discarded in the analysis.

Occasionally, samples presented discontinuities with several distinct SLB patches within the experimentally accessible area (500×500 μm^2) (Fig. S5 F). FRAP measurements carried out on the smaller patches yield lower diffusion coefficients when compared with the same experiment on a complete uniform bilayer (Fig. S5 G) due to edge effects. The substrate can also impact the mobility of lipid molecules when results are compared with free standing bilayers (3). Edges effect can however be neglected when investigating nanoscale texturing effects since the imaging scale is considerably smaller.

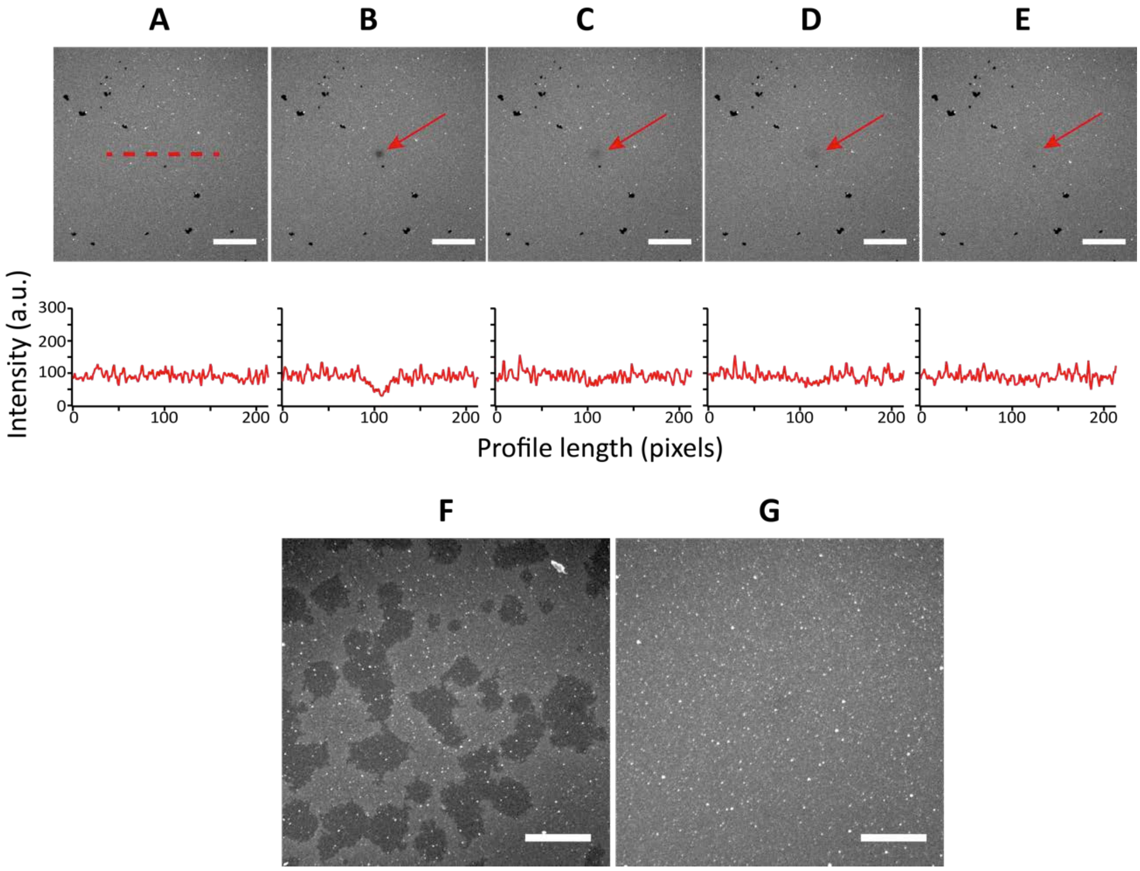

FIGURE S5. Representative fluorescence optical images of a FRAP experiment. An extract of a typical FRAP sequence: image (A) represents the analysed area with the laser off, image (B) is the first frame after bleaching with the bleached area (arrow) clearly visible (see intensity profile). Images (C, D and E) show the gradual fluorescence recovery of the area (arrow). Fluorescence intensity profiles taken across the bleached region are shown below each image. Intensity fluctuations in the profiles are due to the high lipids mobility. Images (F) and (G) illustrate two types of DOPC SLBs (both on mica in solution 7). In (F) the SLB is constituted of bilayer patches while in (G) no patches are visible suggesting a uniform bilayer. All scale bars are 100 µm.

6. REVERSIBILITY OF THE TIP-INDUCED NANO-TEXTURING

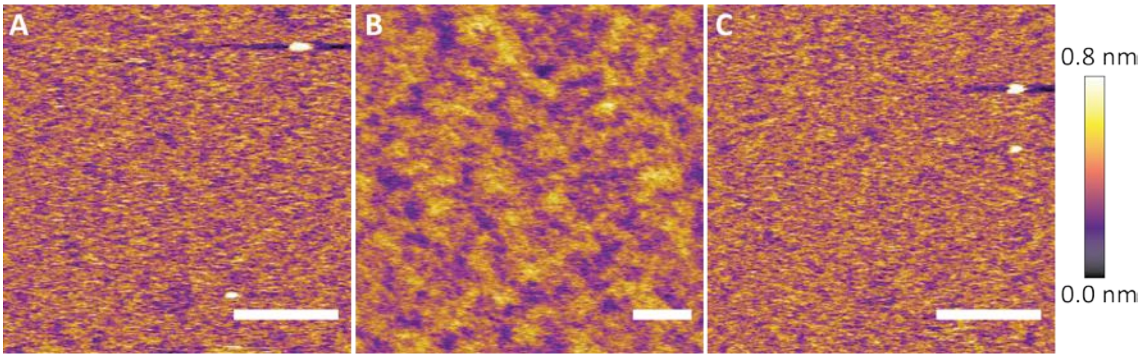

FIGURE S6. AFM images of a DOPC bilayer on mica in solution 6 (KCl 150 mM / $CaCl_2$ 2 mM). First a 1×1 μm^2 area is imaged (A) in the softest condition possible (amplitude 2 nm), followed by a zoomed in image acquired in Hard condition (300×300 nm^2, amplitude 0.6 nm) (B). This is immediately followed by a zoom out image centred over the same area, but acquired again with 2 nm amplitude (1×1 μm^2) (C). No texture or 'memory effect' is visible in the centre of image (C), indicating a relaxation faster than the experimental time (typically 2 minutes). This confirms a high lipid mobility. Scale bars are 300 nm (A, C) and 50 nm (B).

7. CHOLESTEROL STRUCTURING EFFECT

Cholesterol is widely used in model membrane systems due to its importance in natural cell membranes (6). The solubilisation of cholesterol into bilayers influences both fluidity and phase behaviour (4, 7). Cholesterol has a stabilising effect (8-10) on the phospholipids; it modifies the position of lipid tails, and hence the packing arrangement and the overall bilayer thickness.

Here we examine the influence of cholesterol on the stress-induced nano-textured bilayer phase transition observed by AFM. Figure S7 compares AFM images of DOPC bilayer with different concentrations of cholesterol imaged in Hard conditions. Cholesterol is not necessary for nano-texturing of the membrane (Fig. 1), but it clearly reinforces the trend (Fig. S7 C) as to be expected from a molecules promoting gel behaviour in bilayers.

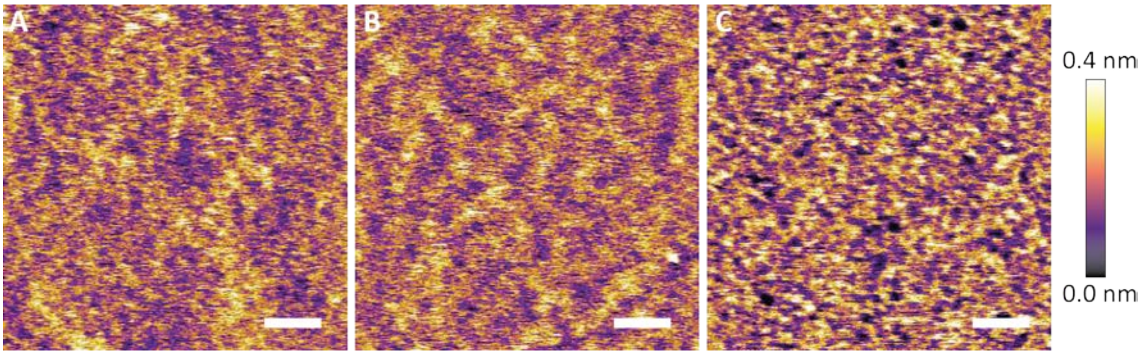

FIGURE S7. Effect of cholesterol on the nano-texturing of DOPC lipid bilayer in solution 1 (KCl 150 mM). The AFM images are taken in Hard imaging condition. Some texture is already visible when no Cholesterol is added (A), but becomes progressively more obvious as 5% relative concentration is added (B) and even more dramatically at 40% concentration (C). Image scale bars are 50 nm.

8. COMPARISON OF IMAGING MODES: CONTACT VS AMPLITUDE MODULATION

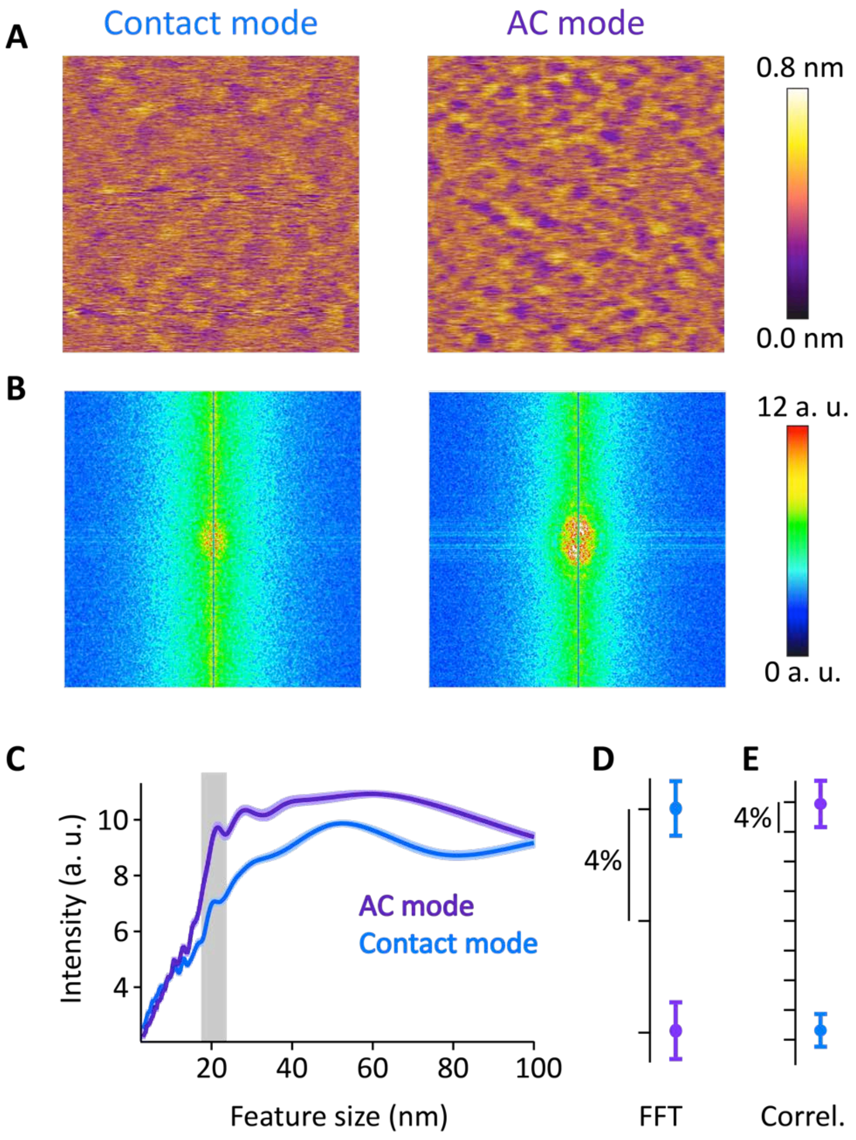

FIGURE S8. Comparison of the features derived from images obtained in contact mode and in amplitude modulation (AM). The contact mode image was obtained in the softest possible conditions, and using the same cantilever as for the AM image (A). The membrane being fluid when not stressed, the contact mode image is challenging and the tip eventually 'ploughs' through the membrane over the following images due to the shear force imposed while scanning. The AM image is obtained in Hard conditions. The 2D FFT images are shown in (B) for both images, on a same colour scale. The average profiles (C, see Fig S4 for explanations) show a maximum around 20 nm for both imaging modes (grey shading) although the maximum is significantly more marked in AM mode. There are also other maxima visible only for the AM image, consistent with a richer variety of features visible in the topographic image. Results of FFT and correlation analysis, conducted as described in the previous section, are given in (D) and (E). Since the analysis is for a single image in each case (and not a difference between Soft and Hard conditions), absolute values are given. The scale represents the different between the two modes, as percentage of the largest value. The FFT analysis (D) shows limited differences between Contact and AM modes (~8%) especially when errors are taken into account. This indicates that long range order exists in both cases. The degree of correlation is however clearly larger (~32%) for the AM images, reflecting a more solid-like membrane under imaging. The scale bar in (A) is 50 nm and the imaging is conducted on a DOPC bilayer immersed in solution 8 (Tris 10 mM / KCl 150 mM / $CaCl_2$ 2 mM).

9. ANALYSIS OF A DOPC BILAYER ON SILICON OXIDE

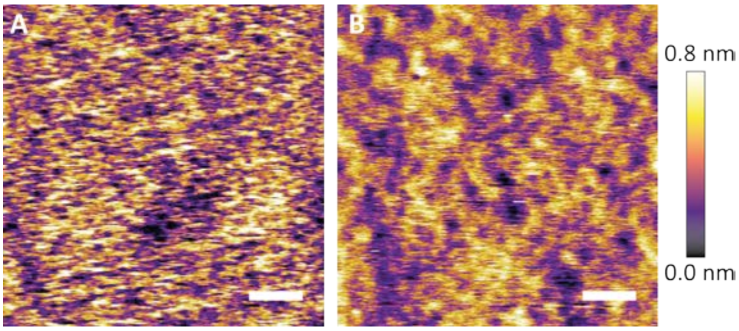

FIGURE S9. AFM analysis of a DOPC SLB formed on a silicon oxide substrate. The cleaned silicon oxide immersed in pure water (A) presents a roughness that is comparable to that of stress-induced nodules in the bilayer, prohibiting any quantitative analysis. The roughness of the DOPC SLB formed on silicon oxide is close to 1 nm, even when using Soft imaging condition (B). The scale bars are 50 nm.

10. EFFECT OF TEMPERATURE ON DOPC WITH SOLUTION 4

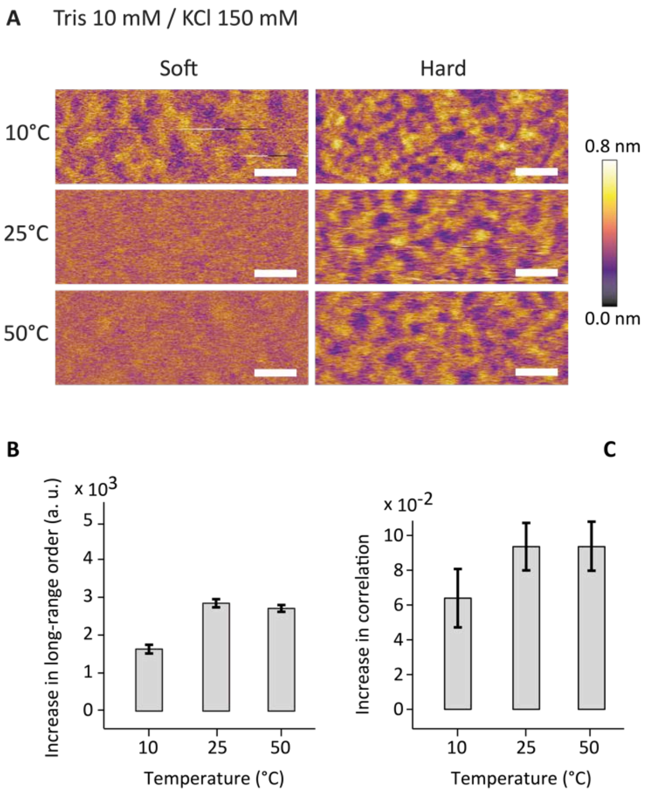

FIGURE S10. AFM images of the evolution of DOPC SLBs on mica as a function of temperature. The experimental is conducted in solution 4 (Tris 10 mM / KCl 150 mM) (A). The graphs in (B) and (C) quantify the evolution of the relative stress-induced nano-texture as a function of temperature. The existence of long-range order is given by FFT analysis (B) while line-by-line correlation (C) quantifies short-range order on the membrane (see Materials and Methods and Fig. S4 for details). In this case, a significant decrease of order is visible at 25 °C and 50 °C in Soft but not in Hard imaging condition. The images scale bar is 50 nm.

11. EFFECT OF TEMPERATURE ON DOPS SLBs

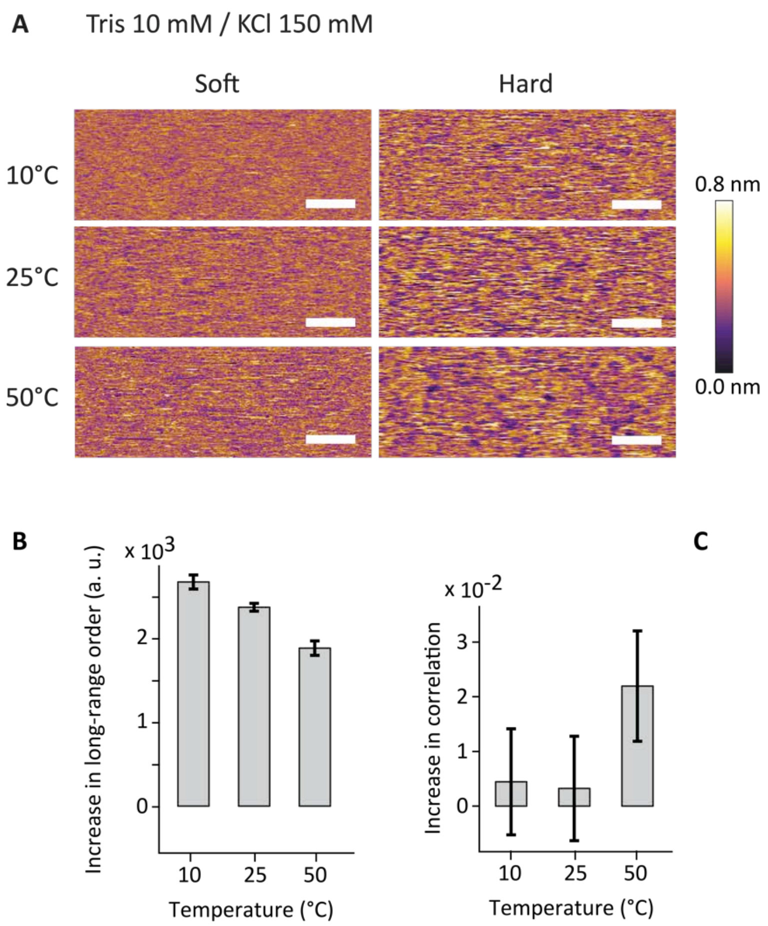

FIGURE S11. Effect of temperature on the stress response of DOPS membranes immersed in solution 4 (Tris 10 mM / KCl 150 mM). The topographic images shown no obvious evolution with temperature although nano-texture tends to be more marked at higher temperatures under Hard conditions. Quantitative analysis confirms this observations with correlation measurements (C) showing some increase at higher temperatures but no clear evolution within error. The increase in long-range order deduced from Fourier analysis (B) shows some decrease with increasing temperature, confirming that DOPS does not react as DOPC and tends to remain fluid throughout the experiment. The images scale bar is 50 nm.

12. COMPARISON OF TEXTURE FORMATION ON DIFFERENT LIPIDS (DOPC AND POPC)

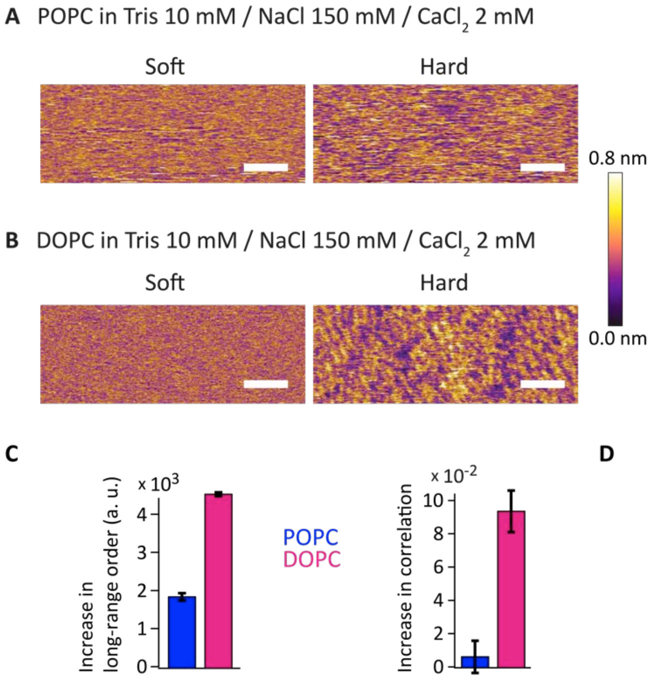

FIGURE S12. Comparison of the stress response of lipid bilayers composed of DOPC (1,2-dioleoyl-*sn*-glycero-3-phosphocholine) and POCP (1-palmitoyl-2-oleoyl-*sn*-glycero-3-phosphocholine) immersed in solution 7 (Tris 10 mM / NaCl 150 mM / $CaCl_2$ 2 mM). The topographic images indicate little change in POPC under stress (A) but significant changes in DOPC under the same conditions (B). This is confirmed by Fourier (C) and correlation (D) analysis of the change between Soft and Hard imaging conditions. The scale bar is 50 nm in (A) and (B).

13. SUPPORTING REFERENCES

1. Mingeot-Leclercq, M. P., M. Deleu, R. Brasseur, and Y. F. Dufrene. 2008. Atomic force microscopy of supported lipid bilayers. *Nat. Protoc.* 3:1654-1659.
2. Blumenthal, D., L. Goldstien, M. Edidin, and L. A. Gheber. 2015. Universal Approach to FRAP Analysis of Arbitrary Bleaching Patterns. *Sci. Rep.* 5:11655.
3. Pincet, F., V. Adrien, R. Yang, J. Delacotte, J. E. Rothman, W. Urbach, and D. Tareste. 2016. FRAP to Characterize Molecular Diffusion and Interaction in Various Membrane Environments. *Plos One.* 11:e0158457.
4. Veatch, S. L., and S. L. Keller. 2003. Separation of liquid phases in giant vesicles of ternary mixtures of phospholipids and cholesterol. *Biophys. J.* 85:3074-3083.
5. Müller, D. J., M. Amrein, and A. Engel. 1997. Adsorption of biological molecules to a solid support for scanning probe microscopy. *J. Struct. Biol.* 119:172-188.
6. Veatch, S. L., and S. L. Keller. 2002. Organization in lipid membranes containing cholesterol. *Phys. Rev. Lett.* 89:268101.
7. Stevens, M. M., A. R. Honerkamp-Smith, and S. L. Keller. 2010. Solubility Limits of Cholesterol, Lanosterol, Ergosterol, Stigmasterol, and beta-Sitosterol in Electroformed Lipid Vesicles. *Soft Matter.* 6:5882-5890.
8. Ma, Y., S. K. Ghosh, D. A. DiLena, S. Bera, L. B. Lurio, A. N. Parikh, and S. K. Sinha. 2016. Cholesterol Partition and Condensing Effect in Phase-Separated Ternary Mixture Lipid Multilayers. *Biophys. J.* 10:1355-1366.
9. Almeida, P. F. 2011. A simple thermodynamic model of the liquid-ordered state and the interactions between phospholipids and cholesterol. *Biophys. J.* 100:420-429.
10. Gumi-Audenis, B., L. Costa, F. Carla, F. Comin, F. Sanz, and M. I. Giannotti. 2016. Structure and Nanomechanics of Model Membranes by Atomic Force Microscopy and Spectroscopy: Insights into the Role of Cholesterol and Sphingolipids. *Membranes (Basel, Switz.).* 6.
